# Physiological and transcriptomic analyses provide insight into thermotolerance in desert plant *Zygophyllum xanthoxylum*

**DOI:** 10.1186/s12870-022-04024-7

**Published:** 2023-01-05

**Authors:** Wan-Peng Bai, Hu-Jun Li, Shelley R. Hepworth, Hai-Shuang Liu, Lin-Bo Liu, Gai-Ni Wang, Qing Ma, Ai-Ke Bao, Suo-Min Wang

**Affiliations:** 1 State Key Laboratory of Herbage Improvement and Grassland Agro-Ecosystems; College of Pastoral Agriculture Science and Technology, Lanzhou University, Lanzhou, 730020 People’s Republic of China; 2 Department of Biology, Institute of Biochemistry, Carleton University, Ottawa, ON Canada

**Keywords:** Thermotolerance, Photosynthesis, RNA-seq, Heat shock transcription factors, Heat shock protein, *Zygophyllum xanthoxylum*

## Abstract

**Background:**

Heat stress has adverse effects on the growth and reproduction of plants. *Zygophyllum xanthoxylum*, a typical xerophyte, is a dominant species in the desert where summer temperatures are around 40 °C. However, the mechanism underlying the thermotolerance of *Z*. *xanthoxylum* remained unclear.

**Results:**

Here, we characterized the acclimation of *Z*. *xanthoxylum* to heat using a combination of physiological measurements and transcriptional profiles under treatments at 40 °C and 45 °C, respectively. Strikingly, moderate high temperature (40 °C) led to an increase in photosynthetic capacity and superior plant performance, whereas severe high temperature (45 °C) was accompanied by reduced photosynthetic capacity and inhibited growth. Transcriptome profiling indicated that the differentially expressed genes (DEGs) were related to transcription factor activity, protein folding and photosynthesis under heat conditions. Furthermore, numerous genes encoding heat transcription shock factors (HSFs) and heat shock proteins (HSPs) were significantly up-regulated under heat treatments, which were correlated with thermotolerance of *Z. xanthoxylum*. Interestingly, the up-regulation of PSI and PSII genes and the down-regulation of chlorophyll catabolism genes likely contribute to improving plant performance of *Z*. *xanthoxylum* under moderate high temperature*.*

**Conclusions:**

We identified key genes associated with of thermotolerance and growth in *Z. xanthoxylum,* which provide significant insights into the regulatory mechanisms of thermotolerance and growth regulation in *Z*. *xanthoxylum* under high temperature conditions*.*

**Supplementary Information:**

The online version contains supplementary material available at 10.1186/s12870-022-04024-7.

## Background

Human-induced global warming has adversely impacted terrestrial ecosystems and contributed to desertification and land degradation in many regions [1–3]. Heat stress due to global warming is a critical threat to crop production and sustainable agriculture world-wide [4]. Therefore, exploring the mechanisms by which temperature regulates plant growth and development will facilitate the breeding of new crop varieties that tolerate high temperature to improve food security [5]. As a whole, heat stress commonly inflicts direct and indirect damage to cells in varying degrees, including the destruction of biomembrane integrity, protein denaturation, and excessive accumulation of reactive oxygen species (ROS) [6, 7]. To cope with heat stress injury, plants have developed complex and efficient mechanisms, involving alterations at physiological, biochemical, and molecular levels [8].

Heat stress is long known to create morphological damage in plants such as scorching, sunburns on shoots, leaf senescence, abscission and growth inhibition, leading to less biomass and reduced yield [7, 9]. Physiologically, the plasma membrane is a major heat sensing structure of cells [10]. Mechanisms that maintain cell membrane integrity are an important adaptation [7]. The heat-tolerant variety had capacity to protect its membrane from disintegration and thereby avoid the leakage of electrolytes [11]. Maintaining lower osmotic potential, as one of the main physiological adjustment mechanism, helps to sustain plant growth by accumulation of inorganic ions and compatible solutes in the cell sap under heat stress [12]. Photosynthesis, the fundamental basis for carbon accumulation, is very sensitive to high temperature stress [13]. Severe high temperature permanently damages enzyme activities in the electron transport chain, carbon metabolism, and oxygen-evolving complex of photosystems resulting in dysfunction of photosynthesis [14].

Heat shock proteins (HSPs), as molecular chaperones, are significantly induced by heat stress to alleviate detrimental effects including the inactivation of enzymes, the denaturation of proteins, and protein aggregation [15]. HSPs can be classified into five classes in plants: HSP100/ClpB, HSP90, HSP70, HSP60 and small HSP (sHSP) [16]. The variation in different HSPs levels is essential for heat acclimation and thermotolerance in plants [17]. Heat shock transcription factors (HSFs) forming a group of central regulators, play critical roles in heat signal transduction pathways, and regulate the expression of HSPs [18, 19].

Although significant progress has been made in the study on molecular and physiological mechanisms of plant heat tolerance in recent years, most studies on heat adaptation have focused on model plants and crops, which are not equally efficient in coping with extreme high temperature [20–22]. Desert plants often converge different superior heat resistance mechanisms, and contains abundant stress resistance gene resources in adaptation during long-term evolution [23]. *Zygophyllum xanthoxylum* Engl., a perennial xerophyte, grows naturally in the arid desert region of northwest China and Central Asia. This species has novel drought and salt stress adaptation strategies and contains rich pools of stress tolerance genes based on physiological analysis and transcriptome profiling [24, 25]. Numerous studies showed that stress tolerance genes of desert plants could facilitate tolerance improvement of crops for drought and salt stresses. For example, overexpression of functional genes from *Cleistogenes songorica* improved tolerance to drought and salt in both alfalfa (*Medicago Sativa*) and Arabidopsis [26, 27]. In previous study, we also demonstrated that co-overexpression of *ZxNHX* and *ZxVP1-1* genes from *Z. xanthoxylum* enhances salt and drought tolerance in alfalfa and *Lotus corniculatus* [28, 29]. Moreover, it is found that *Z. xanthoxylum* is often exposed to high temperature around 40 °C during the growing season, but it can still survive and complete its life cycle well [30, 31], which has aroused our enormous interest. Therefore, we hypothesize that 40 °C may have positive effects on the growth of *Z. xanthoxylum*. To test this possibility, we investigated the phenotypic and physiological responses of *Z. xanthoxylum* when exposed to different high temperature, and then performed an RNA-seq to analyze the differentially expressed genes (DEGs) related to heat shock and photosynthesis, and their potential roles were assessed accordingly. These results provide important clues for further research on molecular mechanisms deployed by desert plants in response to high temperature stress, and such thorough exploration may be highly beneficial to identify suitable genes for biotechnological manipulation to improve thermotolerance of crops.

## Results

### *Z. xanthoxylum* exhibits enhanced thermotolerance under heat treatments

**Fig. 1:**
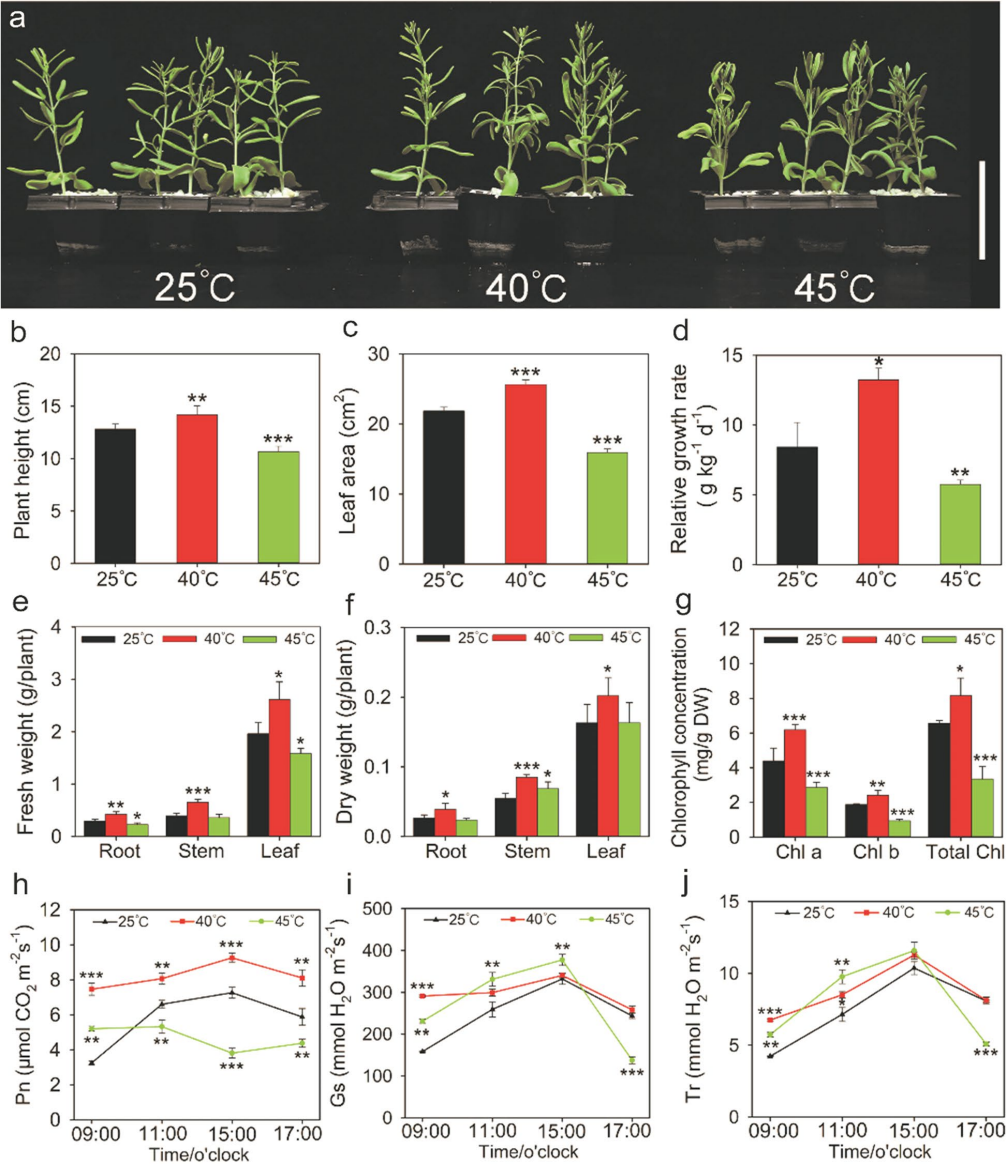
Physiological traits of *Z. xanthoxylum* in response to different heat treatments. (**a**) growth status, (**b**) plant height, (**c**) leaf area, (**d**) relative growth rate, (**e**) fresh weight, (**f**) dry weight, (**g**) chlorophyll concentration, (**h**) net photosynthetic rate (Pn), (**i**) stomatal conductance (Gs) and (**j**) transpiration rate (Tr) of *Z. xanthoxylum* under different heat treatments for 10 days (6 h/d). The time of the beginning of the light phase of the day is 6:00 o’clock. Scale bars in (A) = 10 cm. Values in (**b**-**j**) are the means ± SE (*n* = 5). Asterisks indicate significant differences in comparison with control (25 °C) (* *P* < 0.05, ** *P* < 0.01, *** *P* < 0.001, Student’s *t*-test)

Eighteen-day-old *Z. xanthoxylum* seedlings were subjected to heat treatments for 10 days (6 h/d). Compared with 25 °C, the plants grown at 40 °C were larger and more vigorous (Fig. 1a). Under this condition, the plant height, leaf area and relative growth rate significantly increased by 11%, 17% and 57%, respectively (Fig. 1b-d). Likewise, the fresh and dry weight of root, stem and leaf was significantly increased at 40 °C (Fig. 1e, f). By contrast, *Z. xanthoxylum* showed severe growth retardation at 45 °C, with the above indicators of biomass remarkably decreased (Fig. 1a-e). In addition, chlorophyll (Chl), as one of the important growth related traits, was significantly influenced by heat treatments. The 40 °C treatment caused a significant increase in Chl content, where Chl a and Chl b were 116% and 156% higher, respectively, than in control plants (Fig. 1g). Conversely, Chl a and Chl b were reduced by 35% and 50% at 45 °C relative to 25 °C, respectively (Fig. 1g).

**Fig. 2:**
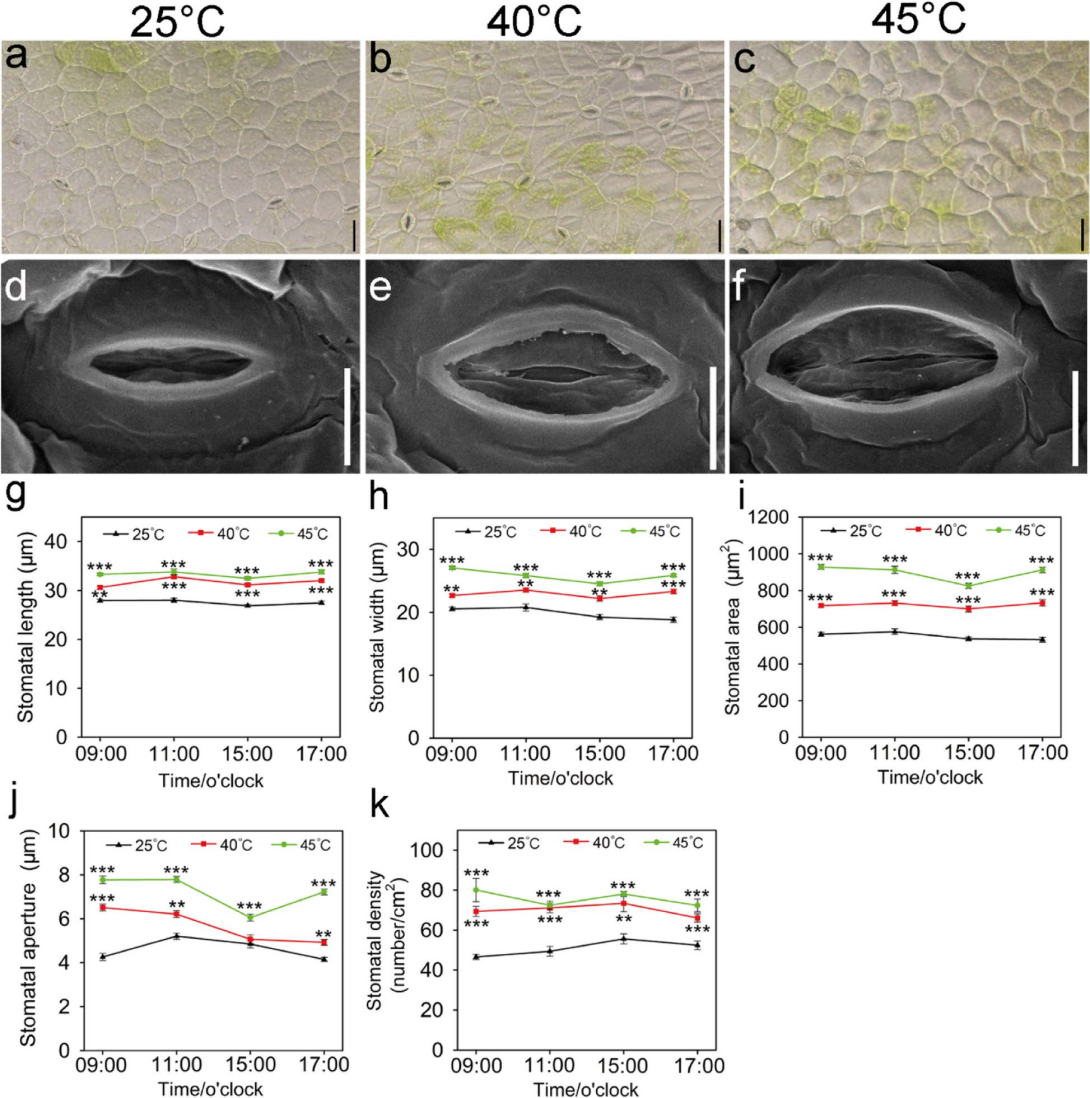
The stomatal morphology and traits in leaves of *Z. xanthoxylum* under control and heat treatments for 10 days (6 h/d). The stomatal distribution (**a**-**c**) and size (**d**-**f**) of leaves at 25 °C (**a**, **d**), 40 °C (**b**, **e**) and 45 °C (**c**, **f**), respectively. Scale bars (**a**-**c**) = 50 μm, Scale bars (**d**-**f**) = 10 μm. The stomatal length (**g**), width (**h**), area (**i**), aperture (**j**) and density (**k**) of leaves were measured. The time of the beginning of the light phase of the day is 6:00 o’clock. Values in (**a**-**i**) are mean ± SE (*n* = 15). Asterisks indicate significant differences in comparison with control (25 °C) (* *P* < 0.05, ** *P* < 0.01, *** *P* < 0.001, Student’s *t*-test)

To investigate possible reasons for the faster growth of *Z. xanthoxylum* under 40 °C moderate heat conditions, the diurnal dynamic changes of net photosynthetic rate (Pn), stomatal conductance (Gs) and transpiration rate (Tr) at 40 °C or 45 °C after 10 days (6 h/d) were examined. In comparison with 25 °C, the Pn, Gs and Tr of plants from 9:00–17:00 at 40 °C was elevated (Fig. 1h-j). During the tested time series, Pn, Gs and Tr at 09:00 h were increased to 130%, 84% and 60%, respectively, in plants treated with 40 °C; in addition, Pn at 15:00 h was reduced to 48%, while Gs and Tr at 09:00 h were increased to 46% and 36% in plants treated with 45 °C compared with control plants, respectively; these were the time points with the greatest differences for each trait between the two groups (Fig. 1h-j). As the primary conduits for gas exchange between plants and the atmosphere, stomata is another important structure affecting the leaf water balance. Therefore, stomatal morphology and traits were observed and evaluated for *Z. xanthoxylum* exposed to different temperatures. The distribution and size of stomata was similarly increased at 40 °C and 45 °C compared with 25 °C (Fig. 2a-f). The stomatal length, width, area, aperture and density significantly increased at 40 °C and 45 °C (Fig. 2g-k).

Maintaining water balance in plants is essential for their survival under high temperature conditions. Therefore, the leaf water content was determined under heat treatments. Compared with 25 °C, there was no significant difference in the water content of leaves at 40 °C, while the leaf water content at 45 °C was significantly reduced (Fig. S1a). In addition, the leaf osmotic potential at 40 °C and 45 °C was significantly decreased by 28% and 42%, respectively (Fig. S1b). whereas the relative membrane permeability was increased by 54% only at 45 °C (Fig. S1c).

### Illumina sequencing, de novo assembly and functional annotation

To explore the genetic response of *Z. xanthoxylum* to heat treatments, the transcriptome was analyzed. Plants were grown at 25 °C and exposed to 40 °C or 45 °C for 0.5 h or 6 h. Roots and leaves were harvested and samples were sequenced in triplicate using independent RNA samples. Each sample was sequenced with more than 46 million raw reads, 42 million clean reads, and 6.36 G of clean bases (Table S1). Moreover, the percentage of clean reads Q20 and Q30 (those with a base quality greater than 20 and 30) were over 97% and 88%, respectively (Table S1). These results suggested that the sequencing output and high quality reads were adequate for further analysis. A total of 166,892 unigenes were obtained from 36 samples with an average length of 1534 bp; the N50 length was 2213 bp, and the percentage of GC was over 40% (Table S2). The size-distribution analysis showed that the lengths of 98,657 unigenes were greater than 1000 bp (Fig. S2a). These results demonstrated the effectiveness of Illumina sequencing in rapidly capturing a large portion of the transcriptome.

To analyze and predict the function of the unigenes, functional annotation was performed. Of 166,892 total unigenes, 139,130 (83.37%) were matched to orthologs in the public databases. Among these, 134,383 (80.52%), 102,235 (61.26%), 105,588 (63.27%), 110,032 (65.93%), 110,642 (66.30%), 103,428 (61.97%) and 104,361 (62.53%) unigenes were found in the NR, NT, Swiss-Prot, KEGG, KOG, Pfam, GO databases, respectively (Table S3). Additionally, all of the unigenes were further assigned to GO databases. A total of 166,892 unigenes were annotated and classified into three main categories: biological processes, cellular components, and molecular functions (Fig. S2b). Binding (52,038), catalytic activity (50,084); cell (29,847), membrane part (29,001), organelle part (12,078); biological regulation (10,934), cellular component or organization or biogenesis (7046), cellular process (28,268), localization (6446), metabolic process (5559), response to stimulus (3202) were the dominant terms in all three categories (Fig. S2b). To further predict unigene function and assess the integrity of the transcriptome, we searched for all unigenes in the KOG database. Based on the KOG database, 110,642 unigenes were classified into 25 functional categories; general function prediction only (20,070) represented the largest group, followed by signal transduction mechanisms (13,133), posttranslational modification, protein turnover, chaperones (10,787) (Fig. S2c).

### Identification and enrichment analysis of DEGs

In leaves of *Z. xanthoxylum*, compared with 25 °C for 0.5 h, 50,486 or 55,440 DEGs were identified at 40 °C or 45 °C, respectively (Fig. S3a). Among these pools, 17,924 and 20,577 DEGs were up-regulated, whereas 32,562 and 34,863 DEGs were down-regulated, respectively (Fig. S3a). Compared with 25 °C for 6 h, 56,011 or 46,979 DEGs were identified in leaves of *Z. xanthoxylum* at 40 °C or 45 °C, respectively (Fig. S3a). Among these pools, 25,320 and 24,931 DEGs were up-regulated and 30,691 and 22,048 DEGs were down-regulated, respectively (Fig. S3a). In roots of *Z. xanthoxylum*, compared with 25 °C for 0.5 h, 37,053 or 45,824 DEGs were identified at 40 °C or 45 °C, respectively (Fig. S3b). Among these pools, 18,076 and 19,602 DEGs were up-regulated, whereas 18,977 and 26,222 DEGs were down-regulated, respectively (Fig. S3b). Compared with 25 °C for 6 h, 62,999 or 61,519 DEGs were identified in roots of *Z. xanthoxylum* at 40 °C or 45 °C, respectively (Fig. S3b). Among these pools, 29,198 and 27,739 DEGs were up-regulated, whereas 33,801 and 33,780 DEGs were down-regulated, respectively (Fig. S3b).

GO enrichment analysis was performed on the DEGs in leaves and roots of *Z. xanthoxylum* treated with 40 °C or 45 °C for 0.5 h or 6 h (Fig. S4; Fig. S5). DEGs were categorized into 57 and 56 GO terms in leaves and roots under heat treatments, respectively (Fig. S4; Fig. S5). Based on the corrected *P*-values, we selected the 20 most-enriched GO terms. Primary terms in this set included “nucleus”, “regulation of transcription, DNA-templated”, “carbohydrate metabolic process”, “DNA-binding transcription factor activity”, “integral of component of membrane”, “protein folding”, “chloroplast”, and “heme binding” (Fig. S4; Fig. S5). To explore the functions of these DEGs, we mapped them to canonical reference pathways in the KEGG database. The DEGs in *Z. xanthoxylum* were assigned to 20 KEGG pathways. Most of these pathways were grouped into “protein process in endoplasmic reticulum”, “plant hormone signal transduction”, “starch and sucrose metabolism” categories in both leaves and roots (Fig. S6; Fig. S7). Based on these results, we further analyzed the response of transcription factors, heat-related proteins and photosynthesis to heat treatments.

### DEGs related to transcription factors

**Fig. 3:**
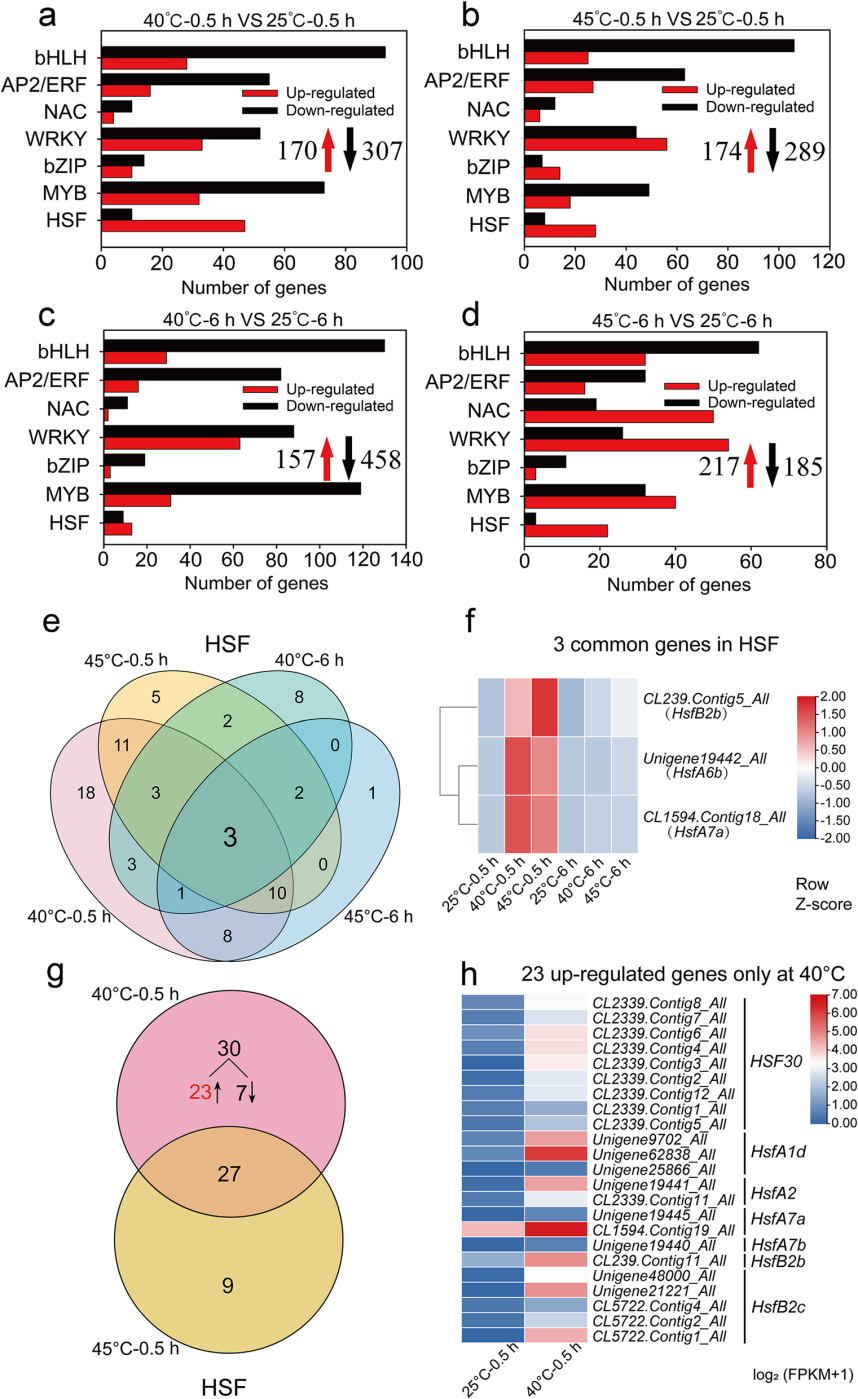
The expression of transcription factor genes in leaves of *Z. xanthoxylum* under different heat treatments. (**a**) and (**b**) show the number of DEGs at 40 °C and 45 °C for 0.5 h, respectively; (**c**) and (**d**) show the number of DEGs at 40 °C and 45 °C for 6 h, respectively. The red upward arrows and black downward arrows show the total number of up-regulated DEGs and down-regulated DEGs, respectively. (**e**) Venn diagrams showing the number of unique and common DEGs related to HSFs in leaves of *Z. xanthoxylum* under heat treatments. (**f**) Heat maps of transcript abundance for three common genes of HSFs in *Z. xanthoxylum* at 40 °C and 45 °C for 0.5 h and 6 h. Gene expression levels were calculated and normalized using the FPKM method. (**g**) Venn diagrams showing the number of unique and common DEGs related to HSFs in leaves of *Z. xanthoxylum* at 40 °C and 45 °C for 0.5 h. (**h**) Heat maps of transcript abundance for 23 up-regulated HSFs identified only at 40 °C for 0.5 h. The relative expression levels were calculated after log_2_ transformation of FPKM values plus 1

Transcription factors (TFs) are important regulators in higher plants. In leaves, TFs including HSF (heat shock transcription factor), bHLH (basic helix-loop-helix), AP2/ERF (APETALA2 and ethylene response factor), bZIP (basic leucine zipper), MYB (myeloblastosis) and WRKY (WRKY-domain) classes were differentially expressed in *Z. xanthoxylum* under heat treatments (Fig. 3a-d). In particular, DEGs encoding HSFs where enriched among up-regulated versus down-regulated genes at 40 °C or 45 °C for 0.5 h or 6 h (Fig. 3a-d). Notably, 3 HSF genes (*HsfA7a*, *HsfA6b* and *HsfB2b*) were up-regulated at 40 °C and 45 °C for 0.5 h and 6 h (Fig. 3e, f), suggesting the consistent expression of these genes performed a core function in cope with high temperature condition. Overall, the number of up-regulated genes encoding HSFs was lower at 40 °C or 45 °C for 6 h (13 or 22 DEGs) compared to 0.5 h (47 or 28 DEGs) (Fig. 3a-d) implying that rapid expression of HSFs plays a protective role during early heat exposure in *Z. xanthoxylum*. After heat treatments for 0.5 h, the number of up-regulated HSF genes at 40 °C (47 DEGs) was much higher than at 45 °C (28 DEGs) (Fig. 3a, b). Among these DEGs, 23 (9 *HSF30*, 3 *HsfA1d*, 2 *HsfA2*, 2 *HsfA7a*, 2 *HsfA7b*, 1 *HsfB2b* and 5 *HsfB2C*) *HSFs* were specific to treatment at 40 °C (Fig. 3g, h).

**Fig. 4:**
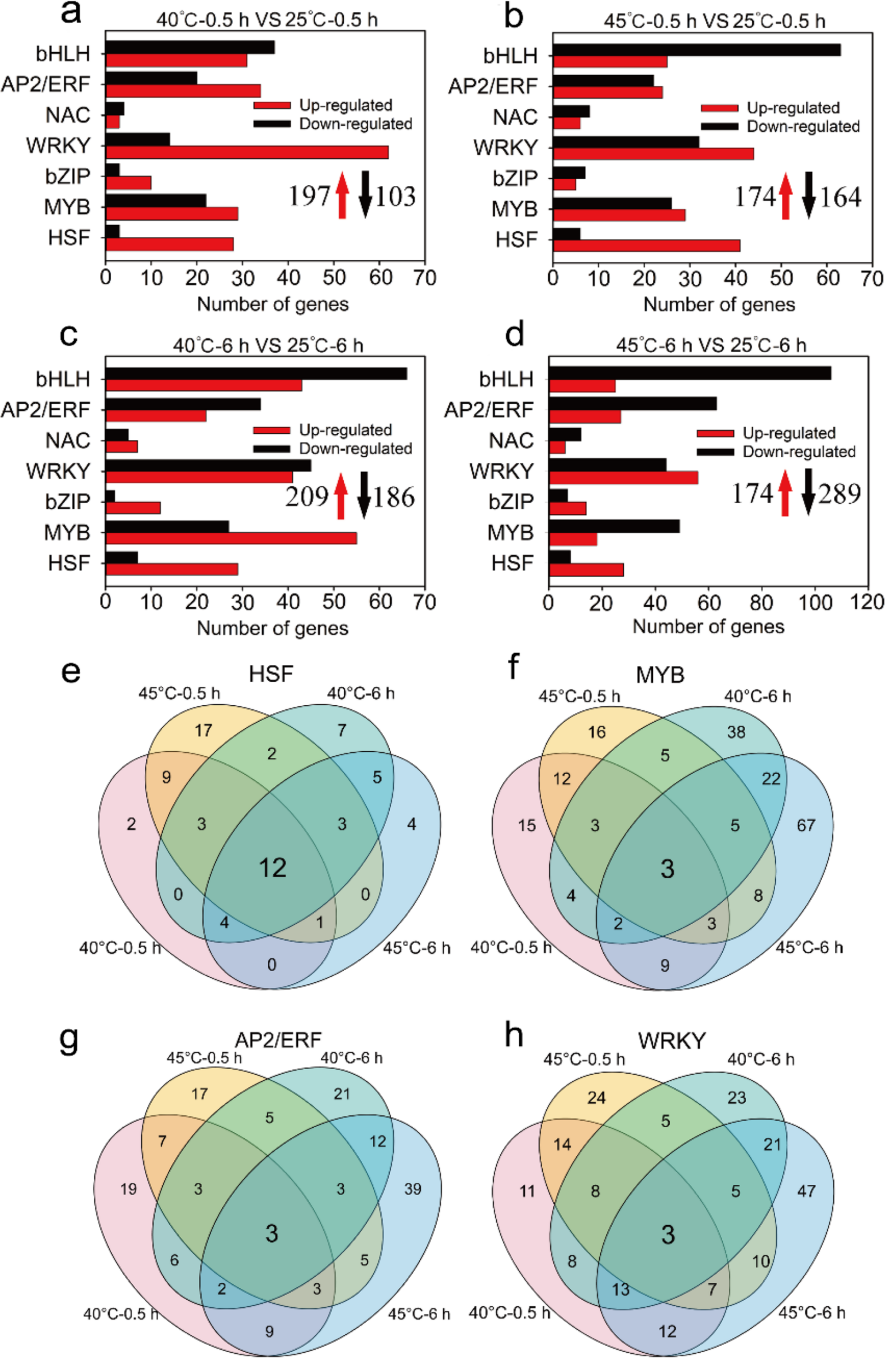
The expression of transcription factor genes in roots of *Z. xanthoxylum* under heat treatments. (**a**) and (**b**) show the number of DEGs at 40 °C and 45 °C for 0.5 h, respectively; (**c**) and (**d**) show the number of DEGs at 40 °C and 45 °C for 6 h, respectively. The red upward arrows and black downward arrows show the total number of up-regulated DEGs and down-regulated DEGs, respectively. Venn diagrams showing the number of unique and common DEGs related to HSFs (**e**), MYBs (**f**), AP2/ERFs (**g**) and WRKYs (**h**) in roots of *Z. xanthoxylum* at 40 °C and 45 °C for 0.5 h and 6 h

In roots, 300 (197 up-regulated and 103 down-regulated) and 338 (174 up-regulated and 164 down-regulated) DEGs encoding TFs were induced at 40 °C for 0.5 h and 6 h; 395 (209 up-regulated and 186 down-regulated) and 507 (242 up-regulated and 265 down-regulated) DEGs were identified at 45 °C for 0.5 h and 6 h (Fig. 4a-d). DEGs encoding HSF and MYB TFs were enriched among up-regulated versus down-regulated genes at 40 °C or 45 °C after 0.5 h or 6 h (Fig. 4a-d). Among these, 12 *HSFs* including 11 up-regulated DEGs (6 *HsfA7a*, 2 *HsfA6b*, 2 *HsfB2b* and 1 *HsfB4*) and 3 *MYBs* including 1 up-regulated DEG (*MYB14*) were identified at 40 °C and 45 °C for 0.5 h and 6 h (Fig. 4e, f; Table S4) suggesting a main role in conferring heat tolerance in *Z. xanthoxylum*. In addition, after heat treatments for 0.5 h, the number of up-regulated AP2/ERF and WRKY DEGs in roots of *Z. xanthoxylum* at 40 °C (34 and 62 DEGs) was much higher than at 45 °C (24 and 44 DEGs) (Fig. 4g, h). 36 (22 up-regulated) and 44 (37 up-regulated) genes related to AP2/ERF and WRKY TFs were observed only at 40 °C, respectively (Fig. 4g, h; Table S5; Table S6). After heat treatments for 6 h, 29 and 20 up-regulated DEGs encoding HSF were observed at 40 °C and 45 °C, respectively (Fig. 4c, d). Among these DEGs, 10 (2 *HsfA2*, 1 *HsfA3*, 2 *HsfA6*, 3 *HsfA7a* and 2 *HsfB2b*) *HSFs* were observed only at 40 °C (Table S7).

### DEGs related to heat shock proteins

Heat shock proteins (HSPs) are a significant class of molecular chaperones in response to heat stress. Therefore, we analyzed the DEGs encoding HSPs.

**Fig. 5:**
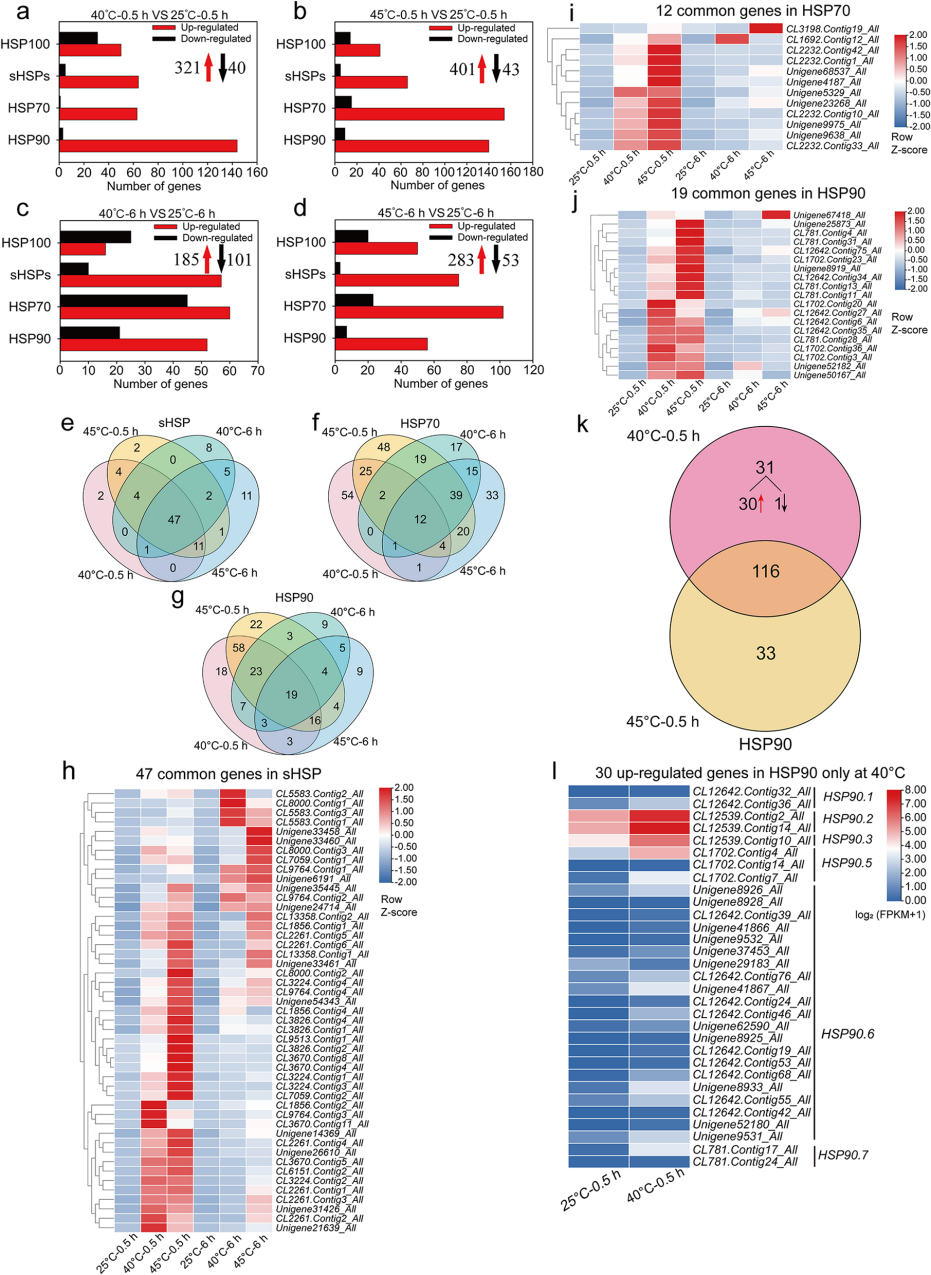
The expression of heat shock protein genes in leaves of *Z. xanthoxylum* under different heat treatments. (**a**) and (**b**) show the number of DEGs at 40 °C and 45 °C for 0.5 h, respectively; (**c**) and (**d**) show the number of DEGs at 40 °C and 45 °C for 6 h, respectively. The red upward arrows and black downward arrows show the total number of up-regulated DEGs and down-regulated DEGs, respectively. Venn diagrams showing the number of unique and common DEGs related to sHSP (**e**), HSP70 (**f**) and HSP90 (**g**) in leaves of *Z. xanthoxylum* under heat treatments. (**h**) Heat maps of transcript abundance for 47 common genes of sHSP in *Z. xanthoxylum* at 40 °C and 45 °C for 0.5 h and 6 h. (**i**) Heat maps of transcript abundance for 12 common genes of HSP70 in *Z. xanthoxylum* at 40 °C and 45 °C for 0.5 h and 6 h. (**j**) Heat maps of transcript abundance for 19 common genes of HSP90 in *Z. xanthoxylum* at 40 °C and 45 °C for 0.5 h and 6 h. Gene expression levels were calculated and normalized using the FPKM method (**h**-**j**). (**k**) Venn diagrams showing the number of DEGs related to HSP90 in leaves of *Z. xanthoxylum* at 40 °C and 45 °C for 0.5 h. (**l**) Heat maps of transcript abundance for 30 up-regulated HSFs identified only at 40 °C for 0.5 h. The relative expression levels were calculated after log_2_ transformation of FPKM values plus 1

In total, 361 (321 up-regulated and 40 down-regulated) and 286 (185 up-regulated and 101 down-regulated) DEGs were categorized as HSPs at 40 °C for 0.5 h and 6 h (Fig. 5a, c). 444 (401 up-regulated and 43 down-regulated) and 336 (283 up-regulated and 53 down-regulated) HSPs were differently expressed at 45 °C for 0.5 h and 6 h in leaves (Fig. 5b, d). The number of up-regulated genes corresponding to sHSP, HSP70 and HSP90 was much higher compared to down-regulated genes in leaves at 40 °C and 45 °C for 0.5 h and 6 h (Fig. 5a-d). Correspondingly, there were 47 (47 up-regulated) sHSP, 12 (11 up-regulated) HSP70 and 19 (17 up-regulated) HSP90 differently expressed genes under heat treatments (Fig. 5e-j). In addition, the number of up-regulated HSP genes in leaves at 45 °C (401 or 283 DEGs) was significantly higher than at 40 °C (321 or 185 DEGs) (Fig. 5a-d), indicating that *Z. xanthoxylum* might accumulate more HSPs to cope with severe high temperature stress. Furthermore, the number of up-regulated HSPs at 40 °C or 45 °C for 0.5 h (321 or 401 DEGs) was far higher than at 6 h (185 or 283 DEGs) (Fig. 5a-d), suggesting that HSPs respond quickly to high temperature in *Z. xanthoxylum*. The number of up-regulated DEGs for HSP90 at 40 °C (144 DEGs) was higher than at 45 °C for 0.5 h (140 DEGs) (Fig. 5a, b). 30 DEGs encoding HSP90s including 2 *HSP90.1*, 2 *HSP90.2*, 1 *HSP90.3*, 3 *HSP90.5*, 20 *HSP90.6* and 2 *HSP90.7* were up-regulated in leaves only at 40 °C (Fig. 5k, l).

**Fig. 6:**
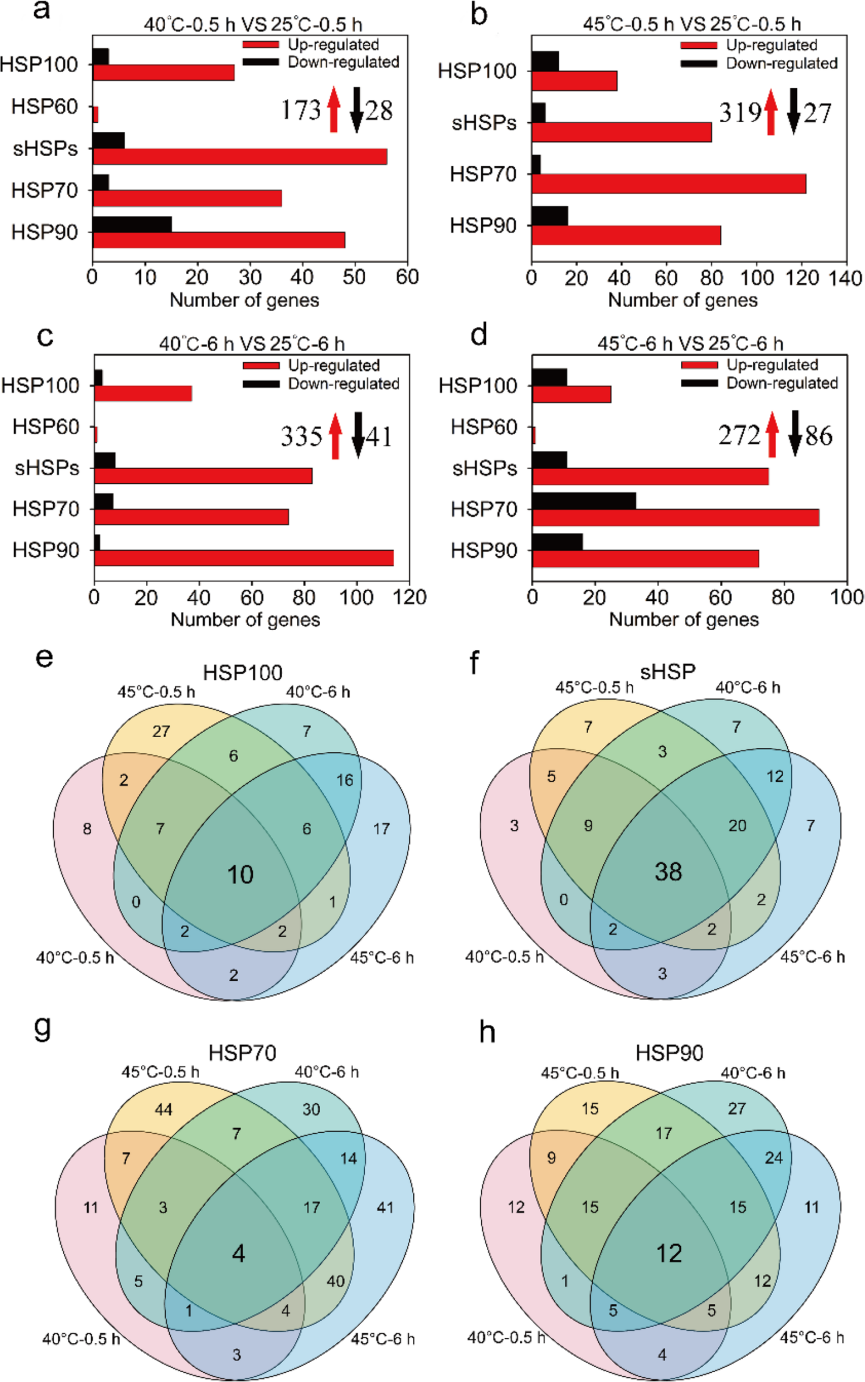
The expression of heat shock protein genes in roots of *Z. xanthoxylum* under heat treatments. (**a**) and (**b**) show the number of DEGs at 40 °C and 45 °C for 0.5 h, respectively; (**c**) and (**d**) show the number of DEGs at 40 °C and 45 °C for 6 h, respectively. The red upward arrows and black downward arrows show the total number of up-regulated DEGs and down-regulated DEGs, respectively. Venn diagrams showing the number of unique and common DEGs related to HSP100 (**e**), sHSP (**f**), HSP70 (**g**) and HSP90 (**h**) in roots of *Z. xanthoxylum* at 40 °C and 45 °C for 0.5 h and 6 h

Similarly, heat treatments caused the significant high expression of DEGs encoding HSPs in roots (Fig. 6a-d). The number of up-regulated genes encoding HSP100, sHSP, HSP70 and HSP90 was much higher compared to down-regulated genes at 40 °C or 45 °C for 0.5 h or 6 h (Fig. 6a-d). Among these DEGs, 10 (9 up-regulated), 38 (38 up-regulated), 4 (4 up-regulated) and 12 (8 up-regulated) corresponding to HSP100, sHSP, HSP70 and HSP90 were differently expressed under heat treatments, respectively (Fig. 6e-h; Table S8). After treatments for 6 h, the number of up-regulated DEGs corresponding to HSP100, sHSP, and HSP90 at 40 °C was much higher than at 45 °C (Fig. 6c, d). Among these, 13 *HSP100*, 19 *sHSP* and 60 *HSP90* genes were identified only at 40 °C (Table S9).

### DEGs related to photosynthesis

Photosynthesis is highly sensitive to heat stress and can reflect plant performance directly. Physiological assays demonstrated that moderate high temperature (40 °C) stimulated the growth of *Z. xanthoxylum*, which was partially related to changes in photosynthesis (Fig. 1h). Thus, we analyzed the DEGs involved in photosynthesis.

**Fig. 7:**
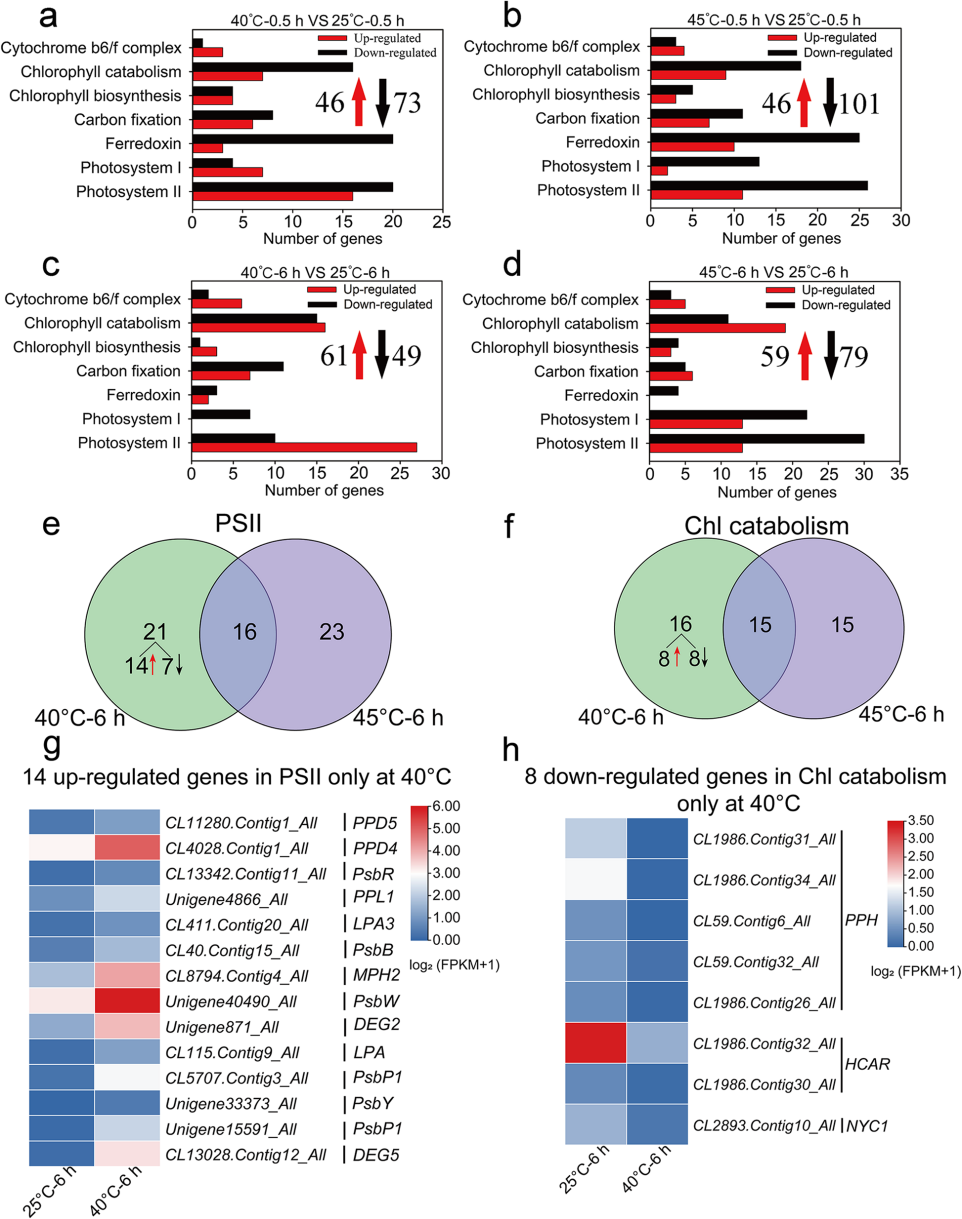
The expression of photosynthesis-related genes in leaves of *Z. xanthoxylum* under different heat treatments. (**a**) and (**b**) show the number of DEGs at 40 °C and 45 °C for 0.5 h, respectively; (**c**) and (**d**) show the number of DEGs at 40 °C and 45 °C for 6 h, respectively. The red upward arrows and black downward arrows show the total number of up-regulated DEGs and down-regulated DEGs, respectively. (**e**) Venn diagrams showing the number of unique and common DEGs related to PSII in leaves of *Z. xanthoxylum* at 40 °C and 45 °C for 6 h. (**f**) Venn diagrams showing the number of unique and common DEGs related to Chl catabolism in leaves of Z. xanthoxylum at 40 °C and 45 °C for 6 h. (**g**) Heat maps of transcript abundance for 14 up-regulated PSII genes identified only at 40 °C for 6 h. (**h**) Heat maps of transcript abundance for 8 down-regulated Chl catabolism genes identified only at 40 °C for 6 h. The relative expression levels were calculated after log_2_ transformation of FPKM values plus 1

In total, 119 (46 up-regulated and 73 down-regulated) or 147 (46 up-regulated and 101 down-regulated) DEGs related to photosynthesis were found in leaves, respectively, at 40 °C or 45 °C for 0.5 h (Fig. 7a, b). The number of down-regulated DEGs involved in photosystem II (PSII), photosystem I (PSI), ferredoxin, Chl biosynthesis and cytochrome b6f complex at 45 °C was much higher than at 40 °C (Fig. 7a, b), suggesting that severe high temperature has pronounced harmful effects on photosynthesis. Of note, the number of up-regulated DEGs (7 DEGs) involved in PSI was much higher than in down-regulated DEGs (4 DEGs) at 40 °C for 0.5 h (Fig. 7a). There were 5 up-regulated DEGs related to PSI only categorized at 40 °C (Fig. 7a; Table S10).

When plants were treated at 40 °C or 45 °C for 6 h, 110 (61 up-regulated and 49 down-regulated) or 138 (59 up-regulated and 79 down-regulated) DEGs were observed, respectively (Fig. 7c, d). Similarly, the number of down-regulated DEGs at 45 °C was much higher than that at 40 °C for 6 h (Fig. 7c, d). Interestingly, after treatments for 6 h, the number of up-regulated DEGs related to PSII and down-regulated DEGs related to Chl catabolism at 40 °C (28 and 15 DEGs) were much higher than at 45 °C (13 and 11 DEGs) (Fig. 7c, d). Among these DEGs, 14 up-regulated DEGs involved in PSII and 8 down-regulated DEGs related to Chl catabolism were only identified at 40 °C (Fig. 7e-h). These results suggest that the changes in DEGs related to PSII and Chl catabolism contribute to the improvement of photosynthesis, which might stimulate the growth of *Z. xanthoxylum* under moderate high temperature.

### Experimental validation

To confirm the reliability of our transcriptome data, 20 isoforms were randomly selected for reverse transcriptase quantitative polymerase chain reaction (RT-qPCR) validation. The RT-qPCR results showed that the expression profiles of these 20 isoforms were highly consistent with their transcript abundance changes determined by RNA-seq. The correlation coefficient *R*^*2*^ of the DEGs from leaves and roots between RT-qPCR and RNA-seq results under heat treatments were over 0.6 (Fig. S8a-h), indicating that our RNA-seq data was accurate and reliable.

## Discussion

### *Z. xanthoxylum* possesses enhanced thermotolerance and moderate high temperature can stimulate its growth

Heat stress, a major limiting factor for plant survival, often causes multifarious and adverse effects on plant growth, development and production [7, 32]. The plant’s response to heat stress varied with intensity, duration and the rate of temperature change [32, 33]. Singh et al. [34] reported that *Lens culinaris* Medik. was able to grow at 27 °C, while grew weakly at temperatures over 35 °C. Nguyen et al. [35] observed a decrease in plant height when sorghum (*Sorghum bicolor*) was subjected to heat stress at 38 °C. *Z. xanthoxylum*, a typical xerophyte, provides excellent genetic resources for improving salt and drought tolerance of crops [28, 29]. Moreover, previous research also showed that *Z. xanthoxylum* experience high temperature over 40 °C in the summer, which greatly exceeds the growth temperature [30]. In the present study, intriguingly, there was no negative effect on the growth of *Z. xanthoxylum* at 40 °C unless the temperature was above to 45 °C (Fig. 1). In addition, there are differences of the optimal growth temperature in various species [36]. A previous study reported that rice and wheat showed optimal growth at 25–30 °C and 20–30 °C, respectively [37]. Attractively, *Z. xanthoxylum* exhibited superior performance with higher biomass and relative growth rate under moderate high temperature (40 °C) (Fig. 1), and such positive phenomenon has not been observed in other studies. By contrast, Wahid et al. [9] showed that the dry weight, growth and net assimilation rates of shoot greatly decreased in sugarcane (*Saccharum officinarum*) at 40 °C. Our results demonstrate that *Z. xanthoxylum* exhibits strong thermotolerance under heat treatments, with optimal growth under moderate high temperature.

### The expression of the genes encoding heat shock transcription factors plays an important role in *Z. xanthoxylum* responding to high temperature

Transcription factors (TFs) are associated with the regulation of genes in response to abiotic stresses like drought, salinity, heat and cold [38]. In perennial ryegrass, many transcription factors (HSFs, AP2/EREBP, MYB, bHLH, and DIVARICATA families) were significantly induced in response to heat stress [39]. Similarly, Bhardwaj et al. [40] showed that numerous TFs belonging to HSFs and DREB families were up-regulated in *Brassica juncea* under high temperature and drought stress. In the present study, several TF families including HSF, bHLH, AP2/ERF, bZIP, MYB and WRKY were induced in leaves and roots of *Z. xanthoxylum* under heat treatments (Fig. 3a-d; Fig. 4a-d). Interestingly, the number of DEGs of TF families was significantly higher in leaves than in roots under short-term high temperature treatments, which may be due to the fact that leaves as the main organ was faster than roots for sensing of the elevate temperature [41]. HSFs are the central regulators of heat stress transcriptome expression in plants [32]. Accordingly, the number of up-regulated HSF genes was much higher among down-regulated genes under heat treatments (Fig. 3a-d; Fig. 4a-d), indicating a significant role for HSFs in response to high temperature in *Z. xanthoxylum*. Previous studies have shown that *AtHSFA7a* knockout mutants have decreased heat stress responsiveness and lose acquired thermotolerance [42, 43]. In addition, HSFA6b, as a downstream regulator of the ABA-mediated stress response, is involved in the heat shock regulation network [21]. In our study, *ZxHsfA7a* and *ZxHsfA6b* were highly induced by heat treatments in both leaves and roots of *Z. xanthoxylum* (Fig. 3e, f; Fig. 4e;Table S4), suggesting that these genes are part of the transcriptional regulation network in heat adaption of *Z. xanthoxylum*.

Furthermore, HSFs play a key role in growth and development of plants. Overexpression of HSFs has resulted in better growth in different plant species under heat stress [44, 45]. In one study, quadruple mutants of *HSFA1a*, *HSFA1b*, *HSFA1d,* and *HSFA1e* exhibited poor performance and weak acquired thermotolerance capacity. Hence, it was suggested that HSFA1s play a pivotal role in heat shock response [46]. Ayako et al. [47] showed that HsfA1d and HsfA1e not only regulate *HsfA2* expression but also function as key regulators of the HSF signaling network in response to heat. Similarly, introducing *AtHsfA1d* into potato could mitigate adverse effects of heat stress, which indicated that HsfA1d plays a vital role in plant thermotolerance [45]. In addition, the high upregulation of HSF30 (belongs to HSFA2) contributes to the thermotolerance of mature tomato microspores and grape [48, 49]. In our study, there were 3 *HsfA1d*, 2 *HsfA2* and 9 *HSF30* genes up-regulated only at 40 °C in leaves (Fig. 3g, h). The expression of these genes may contribute to thermotolerance, thereby improving the growth and development of *Z. xanthoxylum* under moderate high temperature.

### Altered the expression of genes related to heat shock protein contributed to the adaption ability of *Z. xanthoxylum* to high temperature

As one of the significant classes of molecular chaperones, HSPs can protect cellular integrity by inhibiting protein aggregation and providing assistance in refolding and signal transduction as well as participate in the acquisition of thermotolerance [39, 50]. Numerous studies have shown that the expression of most HSPs can be activated in a short time after heat shock [51, 52]. And leaves are often able to sense changes in the surrounding environment more quickly than roots. Likewise, the number of DEGs encoding HSPs was significantly higher in leaves than those in roots under short-term high temperature treatments, indicating that the leaf response to high temperature is more intense in a short time. In leaves of *Z. xanthoxylum*, many HSP encoding genes were strongly up-regulated after heat treatments for 0.5 h, but this number decreased significantly at 6 h (Fig. 5a-d), suggesting that activation of HSPs is a rapid response in coping with high temperatures in leaves. HSP70, HSP90 and sHSP play a primary role in protecting plant cells from the detrimental effects of heat stress [16]. It was demonstrated that the expression of *HSP70-1* correlated with the acquisition of thermotolerance [53, 54]. Another study showed that *Capsicum annuum CaHSP70-2* regulated the expression of genes related to heat stress and conferred increased thermotolerance in Arabidopsis [55]. Yamada et al. [56] showed that HSP90 regulates the heat shock response that is responsible for heat acclimation in Arabidopsis. Overexpression *RcHSP17.8* in Arabidopsis exhibited good viability and increased tolerance to heat, salt and drought stresses [20]. Wang et al. [39] found that *CsHSP17.2* was a heat inducible gene with the capacity to confer thermotolerance in Arabidopsis under heat stress. In this study, a large number of DEGs encoding HSP70, HSP90 and sHSP were up-regulated in leaves and roots of *Z. xanthoxylum* under 40 °C and 45 °C treatments (Fig. 5e-j; Fig. 6; Table S8). These results implicate the induction of *ZxHSP70*, *ZxHSP90* and *ZxsHSP* is important for the adaptation to heat in *Z. xanthoxylum*.

Previous studies reported that HSP90 is key for normal growth and development in Arabidopsis and *Nicotiana benthamiana* [57, 58]. Further, overexpression of *OsHSP90.2* in *E. coli* could enhance cell viability and significantly improve resistance to heat stress [59]. In the current study, 2 *HSP90.2* transcripts (*CL12539.Contig2* and *CL12539.Contig14*) were identified only at 40 °C in leaves (Fig. 5k, l), indicating that high expression of these genes might result in enhanced heat tolerance and superior growth of *Z. xanthoxylum* under moderate high temperature. Previous studies found that overexpression of *HSPs* significantly enhanced thermotolerance by alleviating the damage of heat to photosynthesis [18, 60]. *AtHSP90.5* was predicted to express in the chloroplast, and was mildly induced by heat shock [61]. Cao et al. [62] found that AtHSP90.5 predominately functions in chloroplast biogenesis of cyanobacteria. In this study, 3 *HSP90.5* transcripts (*CL1702.Contig4*, *CL1702.Contig14* and *CL1702.Contig7*) were up-regulated in leaves only at 40 °C (Fig. 5k, l). These results imply that HSP90.5 might participate in the growth of *Z. xanthoxylum* via chloroplast formation for carrying out photosynthesis under moderate high temperature.

### Moderate high temperature stimulates the growth of *Z. xanthoxylum* by improving photosynthesis

Photosynthesis is one of the most sensitive physiological activities of plants to heat stress [13]. Stomatal limitation and non-stomatal limitation are responsible for changes in photosynthetic capacity [63]. Stomatal limitation is mainly caused by the reducing of Gs, which leads a decreased rate of CO_2_ assimilation [64]. We found that while Pn significantly decreased, Gs sharply increased (Fig. 1h-i). This result indicates that non-stomatal limitation is the major reason for photosynthesis reduction in *Z. xanthoxylum* under severe high temperature. Non-stomatal limitations include the reduced activities of carbon fixation enzymes, inhibition of photosystems and maximum electron transport [65]. In this study, a large number of genes related to photosynthesis (especially PSI and PSII) in leaves at 45 °C were down-regulated (Fig. 7b, d), which resulted in photosynthesis reduction. Our results suggest that growth inhibition of *Z. xanthoxylum* under severe high temperature is, at least partly, due to the down-regulation of genes related to PSI and PSII.

However, there was a positive correlation between photosynthesis and temperature during the optimal temperature range (20–30 °C) in plants [66]. In this study, Pn significantly increased at 40 °C relative to 25 °C (Fig. 1h). Moreover, many DEGs related to photosynthesis were up-regulated at 40 °C, especially PSI- and PSII-related genes (Fig. 7a, c). PsaC, PsaE and PsaO are subunit of PSI in eukaryotes, which involved in the electron transport process and regulation of PSI activity [67–69]. Especially, the lack of *PsaE* negatively influences the photosynthesis and growth in plants [68]. In this study, 1 *PsaC* (*CL7285.Contig3_All*), 2 *PsaE* (*CL6683.Contig6_All Unigene48631_All*) and 2 *PsaO* (*Unigene22965_All* and *CL984.Contig8_All*) transcripts were up-regulated only at 40 °C for 0.5 h (Fig. 7a; Table S10), implying that these genes might be required for stable accumulation of PSI and efficient electron transfer. Moreover, the PSII reaction center D1 protein is the main target for light-induced damage among PSII proteins [70]. AtDEG5 (protease Do-like 5) is important for efficient turnover of the D1 protein and for protection against photoinhibition [71]. In addition, strong suppression of PsbP (oxygen-evolving enhancer protein) (5–10% wild-type levels) leads to slow photoautotrophic growth and functional defects associated with both the oxidizing and reducing sides of PSII [72]. In this study, 1 *DEG5* (*CL13028.Contig12_All*) and 2 *PsbP* (*CL5707.Contig3_All* and *Unigene15591_All*) transcripts were up-regulated only at 40 °C for 6 h (Fig. 7e, g), suggesting that these genes might play a functional role in the optimization of photosynthetic oxygen evolution.

Chl is a critical for the photosynthetic process that underpins life on earth, and Chl concentration is an important physiological index related to photosynthesis in plants [24, 73]. The decreases of Chl concentration were a consequence of heat stress in plant [74]. We found that the Chl concentration significantly improved, and a large number of Chl catabolism genes were down-regulated at 40 °C (Fig. 1g; Fig. 7c, f, h). Several studies have shown that as essential Chl catabolic enzymes, pheophytinase (PPH), chlorophyll b reductase NYC1 and 7-hydroxymethyl chlorophyll a reductase (HCAR) were involved in the promotion of Chl degradation of leaves [75–78]. Our results showed that *1 NYC1*, *2 HCAR*, and 5 *PPH* genes were down-regulated only at 40 °C (Fig. 7c, f, h), indicating that these genes might play a crucial role in Chl catabolism in leaves of *Z. xanthoxylum*. This finding suggests that the altered expression of photosynthesis-related genes may improve growth by elevating photosynthetic activity in *Z. xanthoxylum* under moderate high temperature.

## Conclusions

In this study, the phenotypic and physiological indices were analyzed, and transcriptomic analyses were performed on *Z. xanthoxylum* seedlings under high temperature conditions. We found that *Z. xanthoxylum* seedlings exhibited stronger heat tolerance via regulating the expression of the genes encoding HSFs and HSPs, thus maintaining protein homeostasis and alleviating the damage caused by high temperature. Furthermore, moderate high temperature improved plant growth, in association with changes in the expression of photosynthesis-related genes. Moreover, we screened and identified the key genes associated with thermotolerance and growth of *Z. xanthoxylum*. In conclusion, these results provide significant insights into the regulatory mechanisms of thermotolerance and growth regulation in *Z*. *xanthoxylum* under high temperature conditions, and lays a foundation for use in future heat-stress studies of *Z*. *xanthoxylum* and enhances the understanding of molecular mechanisms in heat-tolerant plants.

## Materials and methods

### Plant growth conditions and heat treatments

Our study complied with relevant institutional, national, and international guidelines and legislation, and no specific permits were required to collect the plant samples. All methods were carried out in accordance with relevant guidelines and regulations. *Z. xanthoxylum* seeds were collected in June 2017 from the Minqin (38°03′N, 101°49′E; elevation 1371 m) region, which were identified by Minqin desert botanical garden (http://www.nfgrp.cn/data/list/resource_detaillist.html), and were stored in the Key Laboratory of Grassland Livestock Industry Innovation, Ministry of Agriculture and Rural Affairs, Lanzhou, China [79]. A corresponding voucher specimen (Chase 1700 (K)) has been deposited in stored in School of Life Science, Shihezi University [80]. The seeds were rinsed with water to remove debris, soaked in 5% sodium hypochlorite for 7–10 min, rinsed with distilled water 4–5 times, and then germinated for 2 days at 25 °C on the filter paper in the dark. After germination, the seedlings were transplanted to plug holes in plastic containers (5 × 5 × 5 cm, 1 seedling per container) filled with quartz sand, and irrigated with modified 1/2 strength Hoagland nutrient solution [24]. The seedlings were irrigated with this solution every 2 days, and were cultivated in the greenhouse where the temperature was 25/20 °C (day/night), the photoperiod was 16 h/d (light/dark; light intensity approximately 500 µmol m^−2^ s^−1^), and the relative humidity was approximately 50%.

After 18 days, uniform seedlings were selected and used for the three independent experiments. For physiological experiments, the seedlings were randomly divided into three groups with 32 plants per cultivar for each treatment in a growth chamber (BINDER, KBWF720, Germany). The experiment included three treatments: (1) control, 25/20 °C (day/night), light/dark cycle of 16/8 h (for dark: from 22:00–6:00 h, to simulate night), with two times of irrigation per day; (2) 40 °C treatment for 6 h per day (from 11:00–17:00 h, to simulate high temperature conditions that can occur at midday in the wild) with two times of irrigation per day; the remaining conditions were consistent with the control; (3) 45 °C treatment for 6 h per day (from 11:00–17:00 h, to simulate extreme high temperature conditions that can occur at midday in the wild) with two times of irrigation per day; the remaining conditions were consistent with the control. At the end of the treatment period, photosynthesis-related parameters were determined for the seedlings, which were then harvested for measurements and analyses of other physiological parameters (described below). Three biological replicates were included for each treatment time point, including the control group.

For transcriptome experiments, 18-day-old seedlings were similarly divided into three groups, (1) control, grown at 25 °C, (2) high heat treatment, grown at 40 °C, (3) extreme high heat treatment, grown at 45 °C. The leaves (L) and roots (R) in each treatment were collected after 0.5 h and 6 h, respectively. And each tissue and every time point involved three biological replicates. A total 36 samples were immediately frozen in liquid nitrogen and stored at -80 °C until analysis.

### Physiology analysis in *Z. xanthoxylum* under heat treatments

The 18-day-old seedlings were treated with 40 °C and 45 °C for 10 days, the roots, stems, and leaves of whole plants were taken and rinsed in deionized water, and the tissue fresh weights (FW) were estimated. Next, all samples were oven-dried (80 °C) for 3 days, and the dry weight (DW) was measured. The tissue water content was calculated as water content (%) = (FW – DW) / DW × 100%. The relative growth rate (RGR, g kg^−1^ d^−1^) of whole plants was calculated using the formula: RGR = (lnW_f_ – lnW_i_) / (Δ_t_ × 1000), where W_i_ and W_f_ represent ultimate (i.e. before treatment) and original DW (i.e. after treatment), and Δ_t_ is the time (d) between the two measurements [81].

The photosynthetic parameters including net photosynthesis rate (Pn), stomatal conductance (Gs), and transpiration rate (Tr) were measured in the growth chamber after the start of the photoperiod using an automatic photosynthetic measuring apparatus (GFS-3000, Heinz Walz GmbH, Effeltrich, Germany) in a growth chamber. During the measurements, the temperature in the leaf chamber was set at their respective ambient conditions of each measuring point, relative humidity at 50%, photosynthetic photon flux density at 1000 ± 50 μmol m^−2^ s^−1^, and CO_2_ concentration at 420 ± 20 μmol mol^−1^. Other conditions for gas exchange measurement was kept the same as reported in Cui et al. [81].

Leaf chlorophyll (Chl) content was measured spectrophotometrically as described by Cui et al. [82]. Briefly, the fresh leaf samples were extracted by a 1:1 mixture of 80% acetone (v/v) and 95% ethanol (v/v) until samples were almost white. Absorbance was measured at 645 and 663 nm in a UV spectrophotometer (UV-2102C, Unico Instrument Co., Ltd, Shanghai, China). The Chl a and Chl b contents were then calculated according to Inskeep and Bloom [83].

For stomatal density measurements, mature leaves from *Z. xanthoxylum* plants with different treatments were taken. The stomata were observed quickly by transparent gummed tape to tear epidermis from leaves. Numbers of epidermal pavement cells and stomata were visualized using a light microscope. The number of stomata was counted per cm^2^ (at two different regions per leaf) and stomatal area were calculated [84].

For scanning electron microscopy, the samples were collected and directly placed onto the stub and subsequently frozen in liquid nitrogen shortly. Then, the adaxial surfaces of leaves were observed quickly by scanning electron microscope at accelerating voltage of 15 kV (S-3400 N; Hitachi, Tokyo, Japan).

For leaf osmotic potential, the leaf samples were briefly frozen in liquid nitrogen, and the sap squeezed out from the thawed leaf at 25 °C was detected using the a cryoscopic osmometer (Osmomat-030, Gonotec GmbH, Berlin, Germany). The reading (n, mmol kg^−1^) was used to calculate the Ψs (MPa) using formula: Ψs =–n × R × T, where R = 0.008314 and T = 298.8.

The leaf relative membrane permeability was assessed using a conductivity meter (EC215, HANNA, Italy) as described by Cui et al. [81] as (E1 / E2) × 100, where E1 was the original conductivity of the deionized water with fractured fresh leaves at 25 °C, E2 was the conductivity of the boiled deionized water with fractured leaves.

### Transcriptome sequencing and differentially expressed genes (DEGs) analysis

To obtain the transcriptome of leaves and roots of *Z. xanthoxylum*, total RNA was extracted from leaves and roots using TRIzol® Reagent (Tiangen Biotech, Beijing, China) as previously described [25]. Sequencing libraries were generated as described by Luo et al. [85]. Briefly, the extracted RNA was enriched using magnetic beads (dT) with Oligo. Then, fragmentation buffer was used to fragment the enriched mRNA into short fragments, and reversed the transcription to a double-strand cDNA (dscDNA) with random hexamer (N6) primers. The ends of the dscDNA were repaired with phosphate at the 5′ end and sticky ‘A’ at the 3′ end, then the dscDNA strands were attached with adapters with a sticky ‘T’ at the 3′ end. The ligation products were amplified by PCR using specific primers. Finally, The PCR products were thermal-denatured to single strand DNA, and was cyclized by a bridge primer to obtain a single strand circular DNA library. Subsequently, 36 cDNA libraries were constructed and the cDNA was sequenced on a DNBSEQ platform at BGI Beijing. The raw data were obtained as previously described [85] by removing reads containing adaptors, reads with more than 5% ambiguous bases (“N”), and low-quality reads by Illumina sequencing was analyzed by SOAPnuke (v 1.40). Trinity software (v 2.0.6) was used to assemble the sequences after high-quality clean data were selected. The gene expression level was quantified by the RSEM software (v 1.2.8) package [86] and was normalized by the FPKM method [87]. Gene function were annotated based on Nr (NCBI non-redundant protein database) (http://www.ncbi. nlm.nih.gov/), Nt (NCBI non-redundant nucleotide sequences) (http://www.ncbi. nlm.nih.gov/), Swiss-Prot (http://www.expasy.ch/sprot/), KEGG (the Kyoto Encyclopedia of Genes and Genomes pathway database) (http://www.genome.jp/kegg/), KOG/COG (Cluster of Orthologous Groups database) (http://www.ncbi.nlm.nih.gov/COG), Pfam (http://pfam.xfam.org/) and GO (Gene Ontology) (http://www.geneontology.org) [88–90]. DESeq2 software was used for analysis of DEGs, the false discovery rate (FDR) was used to determine the threshold *P*-value in multiple tests. A FDR < 0.001 and an absolute value of the log_2_ (fold change) > 2 was used as the threshold to determine DEGs [25].

### Reverse transcriptase-quantitative PCR (RT-qPCR) validation

A random selection of 20 genes from *Z. xanthoxylum* were used for the verification of RNA-seq data using RT-qPCR (Table S11). The same RNA and cDNA used as for the RNA sequencing experiments Total RNA was extracted from the 36 samples as described before. Briefly, we used 2 μg of total RNA and followed the manufacturer’s protocol to generate cDNA (TaKaRa, Japan). RT-qPCR was performed using SYBR Green Master Mix (TaKaRa, Japan) on a 7500 Real-time PCR Thermocycler System (ABI, USA). *ZxACTIN* (GenBank accession no. EU019550) was used as the internal control gene [79]. The qRT-PCR analysis included three independent technical repeats with three biological replicates. The relative expression levels were calculated using the 2^−ΔΔCt^ method [89]. Gene-specific primers for RT-qPCR were designed via DNAMAN software (Lynnon BioSoft, Vandreuil, Quebec, Canada) as listed in Table S12.

### Statistical analysis

All data was analyzed using SPSS 19.0 (SPSS Inc., USA). Normal distribution of data was tested using the Kolmogorov–Smirnov test. The statistical analyses were performed using analysis of Student’s *t*-test to detect significant differences between heat treatments and control. All data were presented as mean values ± SE (n ≥ 5). Histograms were performed by SigmaPlot12.5 (Systat Software, Inc., San Jose, CA, USA). Venn diagrams and heat maps were drawn through the TBtools software (v 1.09867) (https://github.com/CJ-Chen/TBtools/releases) [91].

## Supplementary Information

**Additional file 1:** **Figure S1**.The water content (a), osmotic potential (b) and relative membrane permeability(c) of leaves in Z. *xanthoxylum* under control and heat treatments for 10 days(6 h/d). Values in (a-c) are mean ± SE (*n* = 5). Asterisks indicate significantdifferences in comparison with control (25°C) (* *P* < 0.05, ** *P* < 0.01,*** *P* < 0.001, Student’s *t*-test). **Figure S2**. Functional annotation of the assembled transcriptome. (a) Lengthdistribution of all assembled unigenes; (b) Map of GO functional categories; (c) Map of KOG function classifications. **Figure S3**. Number of DEGs in leaves (a) and roots (b) of Z. *xanthoxylum* under heat treatments. **Figure S4**. Enriched GO terms for DEGs in leaves of Z. *xanthoxylum* at 40°C (a, c) and 45°C (b, d) for 0.5 h (a, b) and 6 h (c, d). **Figure S5**. Enriched GO terms for DEGs in roots of Z. *xanthoxylum* at 40°C (a, c) and 45°C(b, d) for 0.5 h (a, b) and 6 h (c, d). **Figure S6**. Enriched KEGG terms for DEGs in leaves of Z. *xanthoxylum* at 40°C (a, c) and 45°C (b, d) for 0.5 h (a, b) and 6 h (c, d). **Figure S7**. Enriched KEGG terms for DEGs in roots of Z. *xanthoxylum*at 40°C (a, c) and 45°C (c, d) for 0.5 h (a, b) and 6 h (b, d). **Figure S8**. Correlation analysis for 20 selected DEGs betweenRNA-seq and RT-qPCR results in leaves (a-d) and roots (e-h) of *Z. xanthoxylum* under heat treatments.

**Additional file 2:** **Table S1**.Overview of RNA sequencing data. **Table S2**. Sequencing production statistics. **Table S3**. Summary of sequence annotation. **Table S4**. DEGs related to MYBs and HSFs in roots of Z. *xanthoxylum* under heat treatments. Fold change equals to log_2_ (the RPKM value of a gene undertreatment / the RPKM value of a gene under control condition) and indicates thetranscript abundance change of each DEGs. Homologous gene and homologous species were obtained by NCBI blast according to the sequence corresponding to the gene ID. **Table S5**. DEGs relatedto AP2/ERFs in roots of Z. *xanthoxylum* identified only at 40°C for 0.5 h. Foldchange equals to log_2_ (40°C-0.5 h RPKM / 25°C-0.5 h RPKM) and indicates thetranscript abundance change of each DEGs. Homologous gene and homologous species were obtained by NCBI blast according to the sequence corresponding tothe gene ID. **Table S6**. DEGs relatedto WRKYs in roots of Z. *xanthoxylum* identified only at 40°C for 0.5 h. Foldchange equals to log_2_ (40°C-0.5 h RPKM / 25°C-0.5 h RPKM) and indicates thetranscript abundance change of each DEGs. Homologous gene and homologousspecies were obtained by NCBI blast according to the sequence corresponding tothe gene ID. **Table S7**. DEGs related to HSFs in roots of Z. *xanthoxylum* identified only at 40°C for 0.5 h. Foldchange equals to log_2_ (40°C-0.5 h RPKM / 25°C-0.5 h RPKM) and indicates thetranscript abundance change of each DEGs. Homologous gene and homologous species were obtained by NCBI blast according to the sequence corresponding tothe gene ID. **Table S8**. DEGs relatedto HSPs in roots of Z. *xanthoxylum* identified under heat treatments. Foldchange equals to log_2_ (the RPKM value of a gene under treatment / the RPKM value of a gene under control condition) and indicates the transcript abundancechange of each DEGs. Homologous gene and homologous species were obtained by NCBI blast according to the sequence corresponding to the gene ID. **Table S9**. DEGs related to HSPs in roots of Z. *xanthoxylum* identified only at 40°C for 6 h. Fold change equals to log_2 _(40°C-6 h RPKM / 25°C-6 h RPKM) and indicates the transcript abundance change of each DEGs. Homologous gene and homologous species were obtained by NCBI blast according to the sequence corresponding to the gene ID. **Table S10**. DEGs related to PSI inleaves of Z. *xanthoxylum* identified only at 40°C for 0.5 h. Fold change equalsto log_2_ (40°C-0.5 h RPKM / 25°C-0.5 h RPKM) and indicates the transcript abundance change of each DEGs. Homologous gene and homologous species were obtained by NCBI blast according to the sequence corresponding to the gene ID. **Table S11**. The sequences used for RT-qPCR validation.

## Data Availability

All the data generated or analysed during this study are included in the manuscript and its additional files. The clean sequencing data have been uploaded to the NCBI Sequence Read Archive (SRA) under the accession number SRR20749110-SRR20749145. The datasets are available from the corresponding author on reasonable request.
