# Supplementary material for: Physiological and transcriptomic analyses provide insight into thermotolerance in desert plant Zygophyllum xanthoxylum
Source: BMC Plant Biol. 2023 Jan 5;23:7. doi: 10.1186/s12870-022-04024-7 (PMC9814312; doi:10.1186/s12870-022-04024-7)
Supplement: Supplementary file 1 — Additional file 1: Figure S1.The water content (a), osmotic potential (b) and relative membrane permeability(c) of leaves in Z. xanthoxylum under control and heat treatments for 10 days(6 h/d). Values in (a-c) are mean ± SE (n = 5). Asterisks indicate significantdifferences in comparison with control (25°C) (* P < 0.05, ** P < 0.01,*** P < 0.001, Student’s t-test). Figure S2. Functional annotation of the assembled transcriptome. (a) Lengthdistribution of all assembled unigenes; (b) Map of GO functional categories; (c) Map of KOG function classifications. Figure S3. Number of DEGs in leaves (a) and roots (b) of Z. xanthoxylum under heat treatments. Figure S4. Enriched GO terms for DEGs in leaves of Z. xanthoxylum at 40°C (a, c) and 45°C (b, d) for 0.5 h (a, b) and 6 h (c, d). Figure S5. Enriched GO terms for DEGs in roots of Z. xanthoxylum at 40°C (a, c) and 45°C(b, d) for 0.5 h (a, b) and 6 h (c, d). Figure S6. Enriched KEGG terms for DEGs in leaves of Z. xanthoxylum at 40°C (a, c) and 45°C (b, d) for 0.5 h (a, b) and 6 h (c, d). Figure S7. Enriched KEGG terms for DEGs in roots of Z. xanthoxylumat 40°C (a, c) and 45°C (c, d) for 0.5 h (a, b) and 6 h (b, d). Figure S8. Correlation analysis for 20 selected DEGs betweenRNA-seq and RT-qPCR results in leaves (a-d) and roots (e-h) of Z. xanthoxylum under heat treatments. [file 12870_2022_4024_MOESM1_ESM.docx]

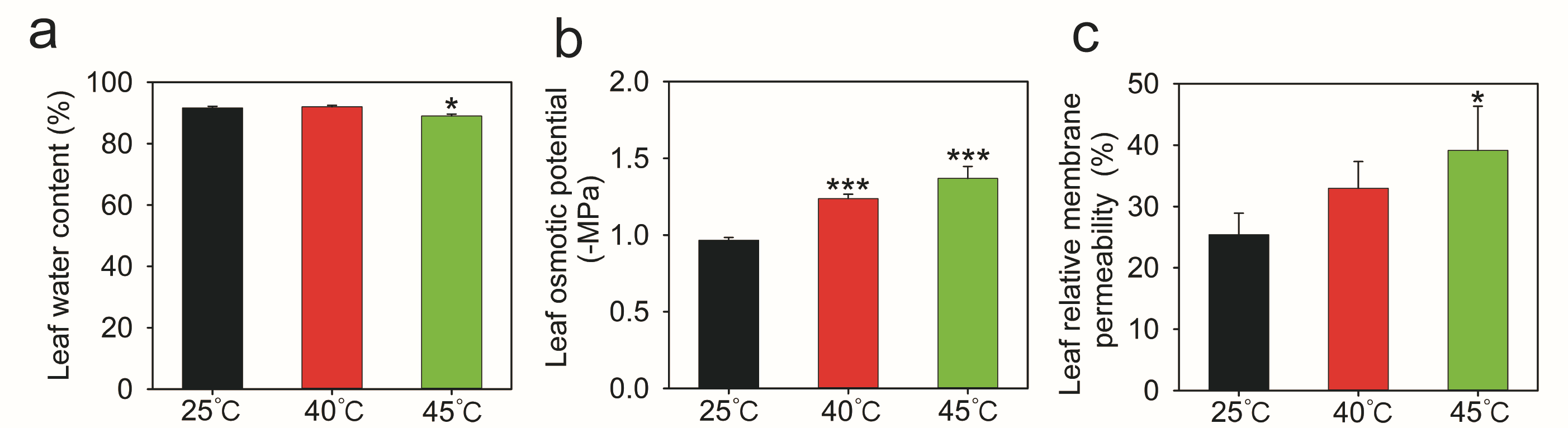


Figure S1. The water content (a), osmotic potential (b) and relative membrane permeability (c) of leaves in *Z. xanthoxylum* under control and heat treatments for 10 days (6 h/d). Values in (a-c) are mean ± SE (n = 5). Asterisks indicate significant differences in comparison with control (25°C) (* *P* < 0.05, ** *P* < 0.01, *** *P* < 0.001, Student’s *t*-test).


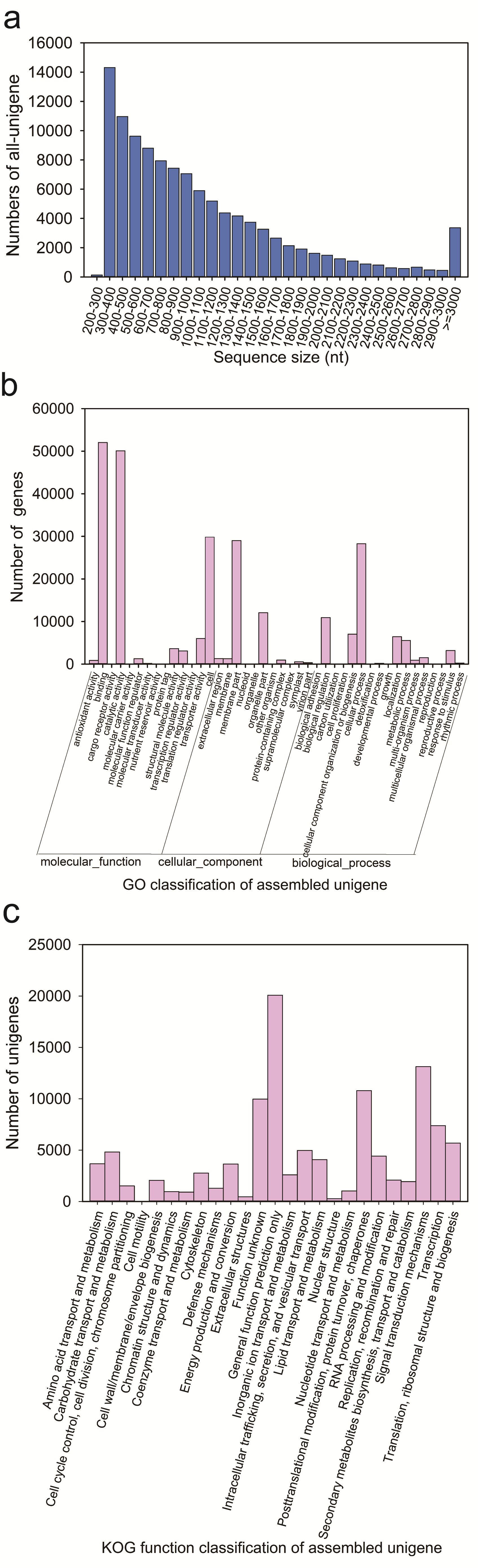


Figure S2. Functional annotation of the assembled transcriptome. (a) Length distribution of all assembled unigenes; (b) Map of GO functional categories; (c) Map of KOG function classifications.


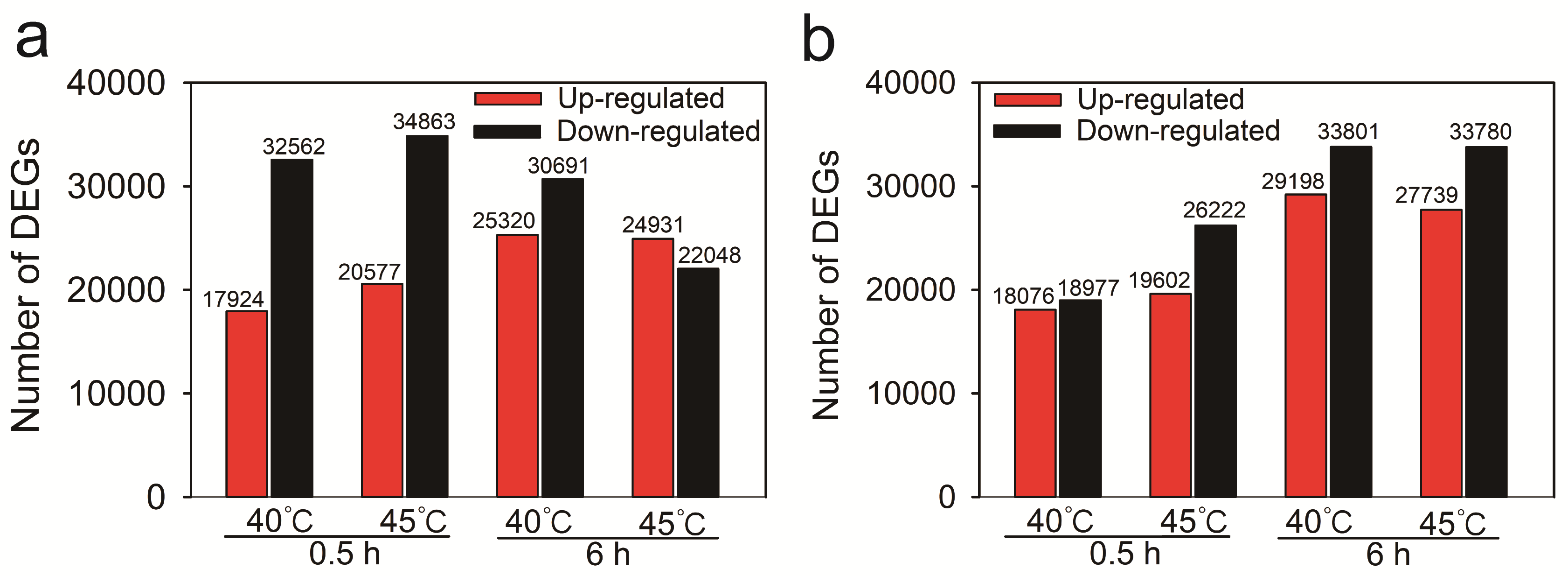


Figure S3. Number of DEGs in leaves (a) and roots (b) of *Z. xanthoxylum* under heat treatments.


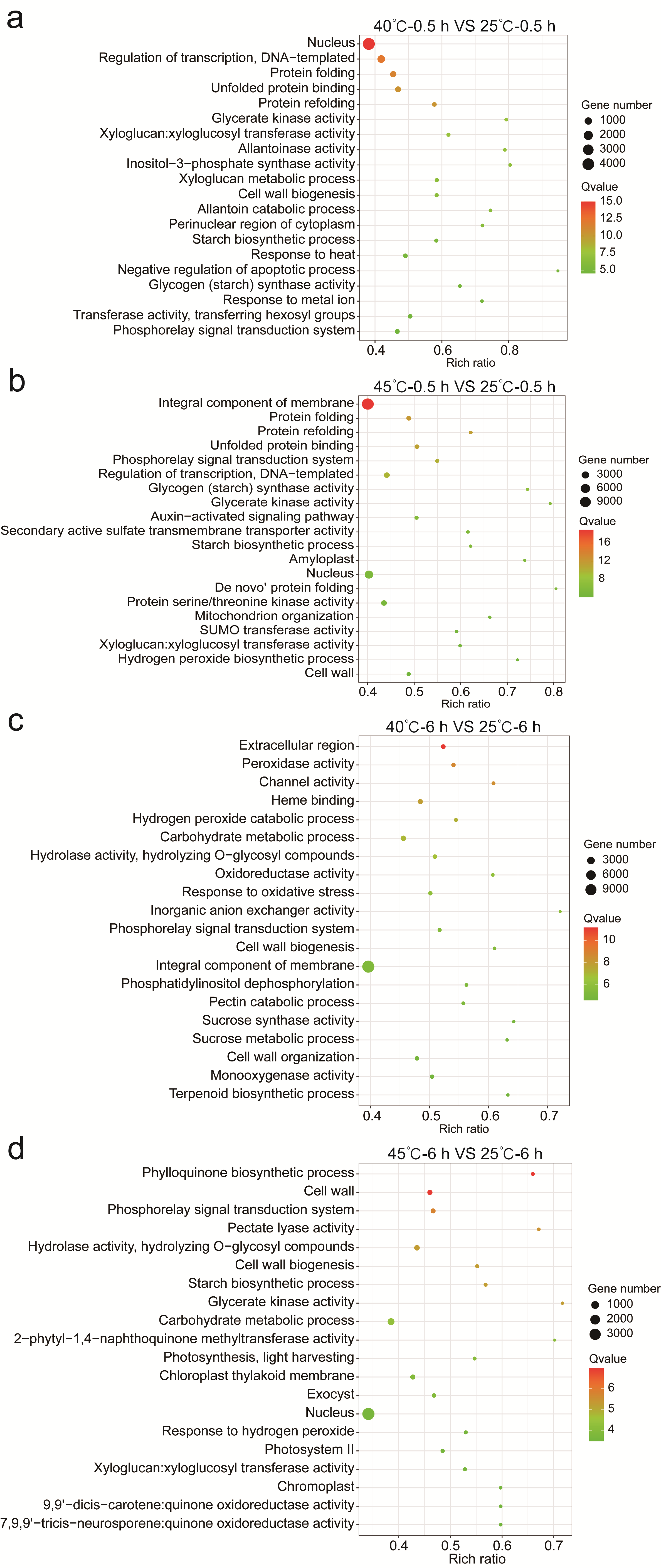


Figure S4. Enriched GO terms for DEGs in leaves of *Z. xanthoxylum* at 40°C (a, c) and 45°C (b, d) for 0.5 h (a, b) and 6 h (c, d).


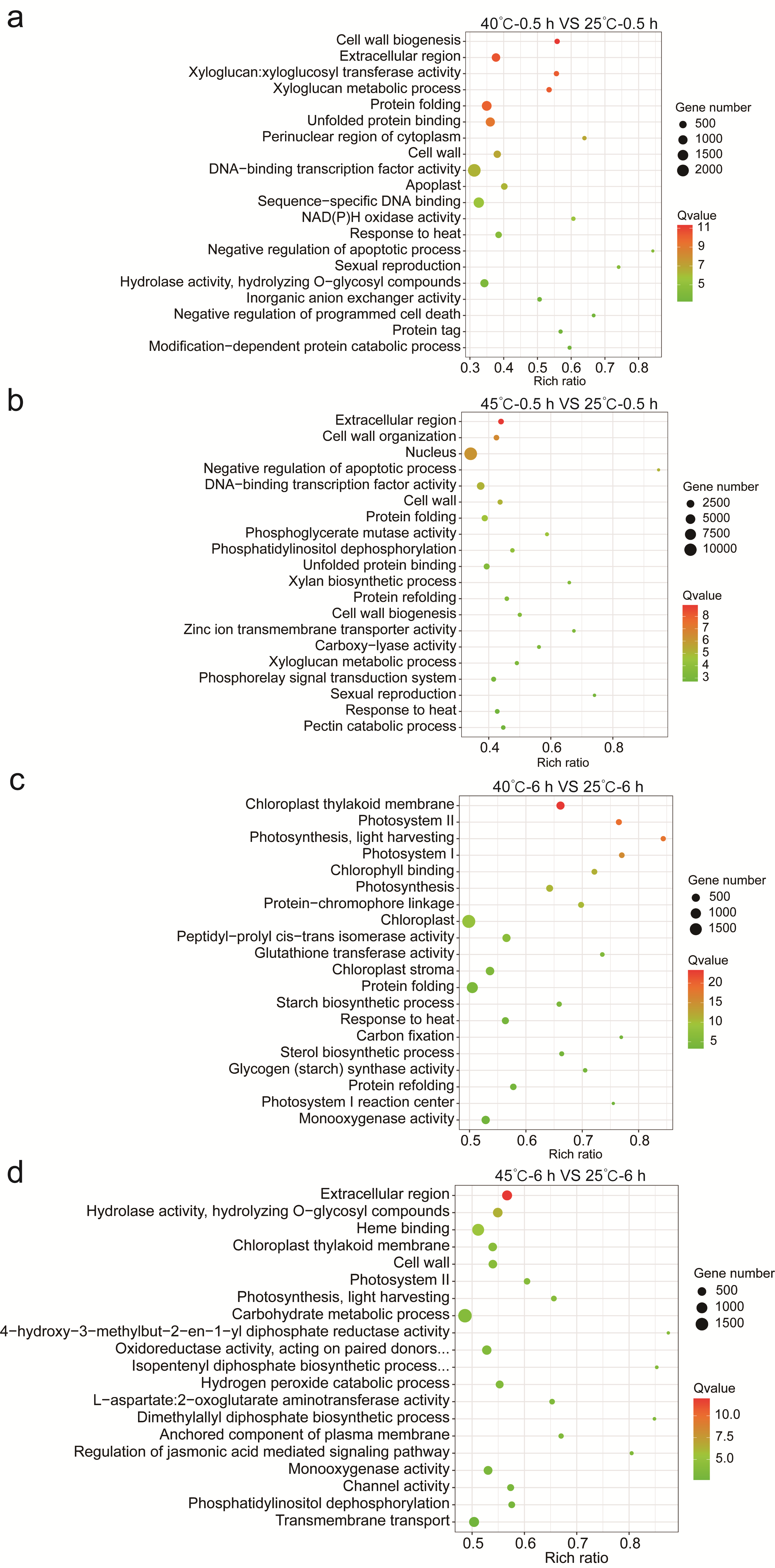


Figure S5. Enriched GO terms for DEGs in roots of *Z. xanthoxylum* at 40°C (a, c) and 45°C (b, d) for 0.5 h (a, b) and 6 h (c, d).


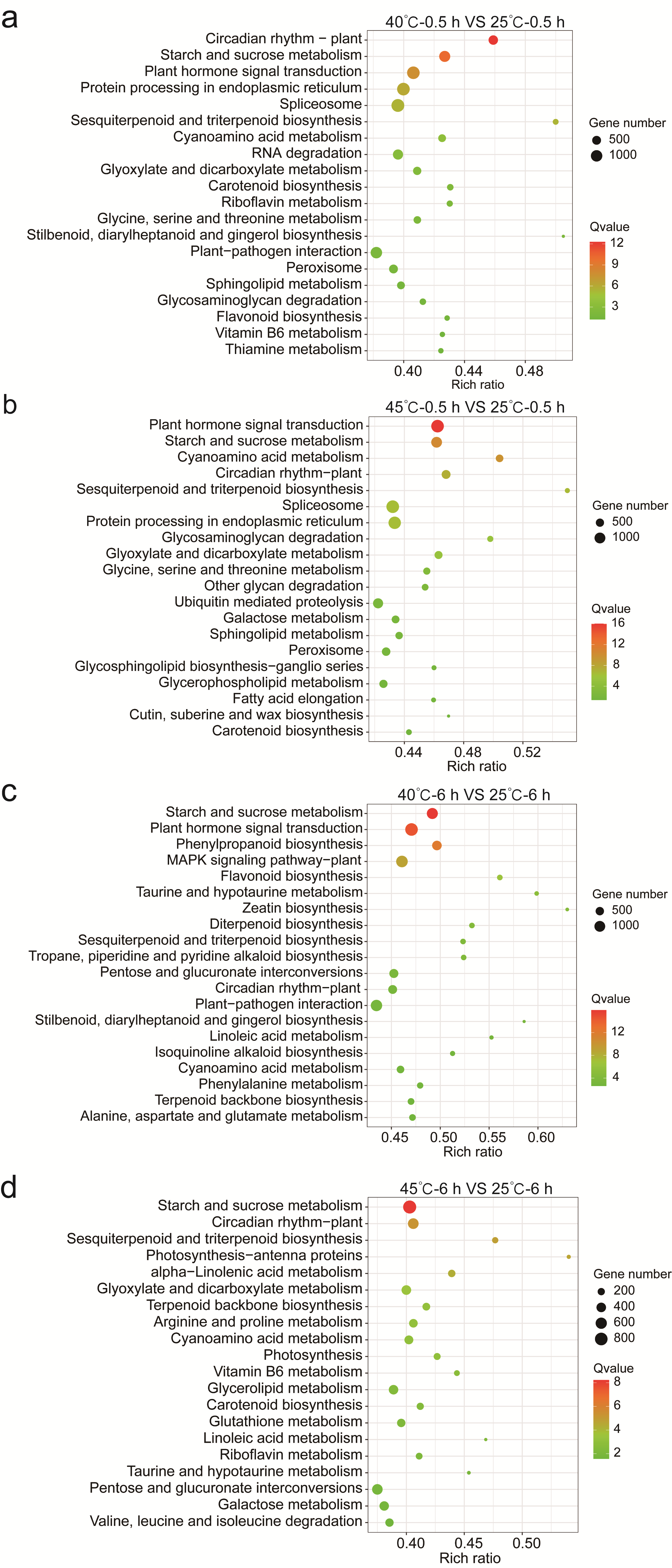


Figure S6. Enriched KEGG terms for DEGs in leaves of *Z. xanthoxylum* at 40°C (a, c) and 45°C (b, d) for 0.5 h (a, b) and 6 h (c, d).


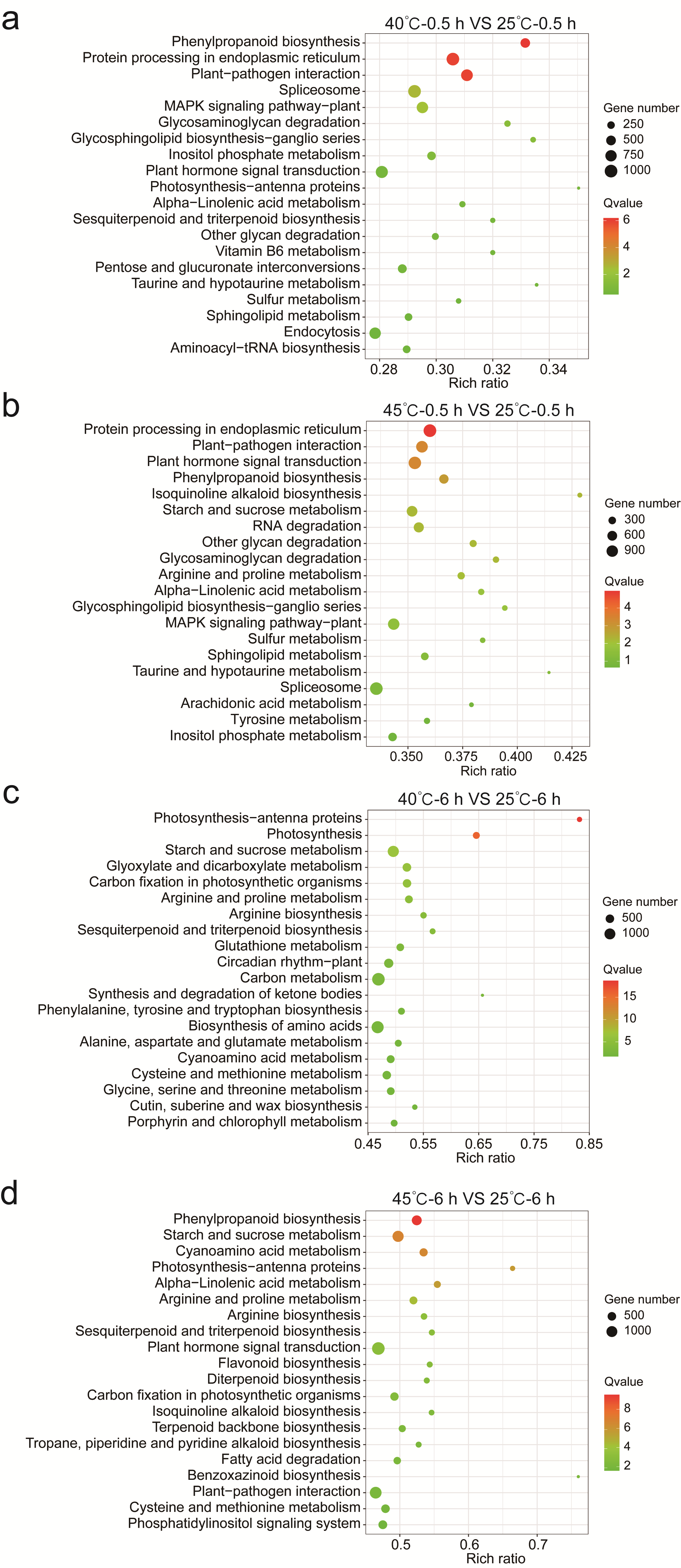


Figure S7. Enriched KEGG terms for DEGs in roots of *Z. xanthoxylum* at 40°C (a, c) and 45°C (c, d) for 0.5 h (a, b) and 6 h (b, d).


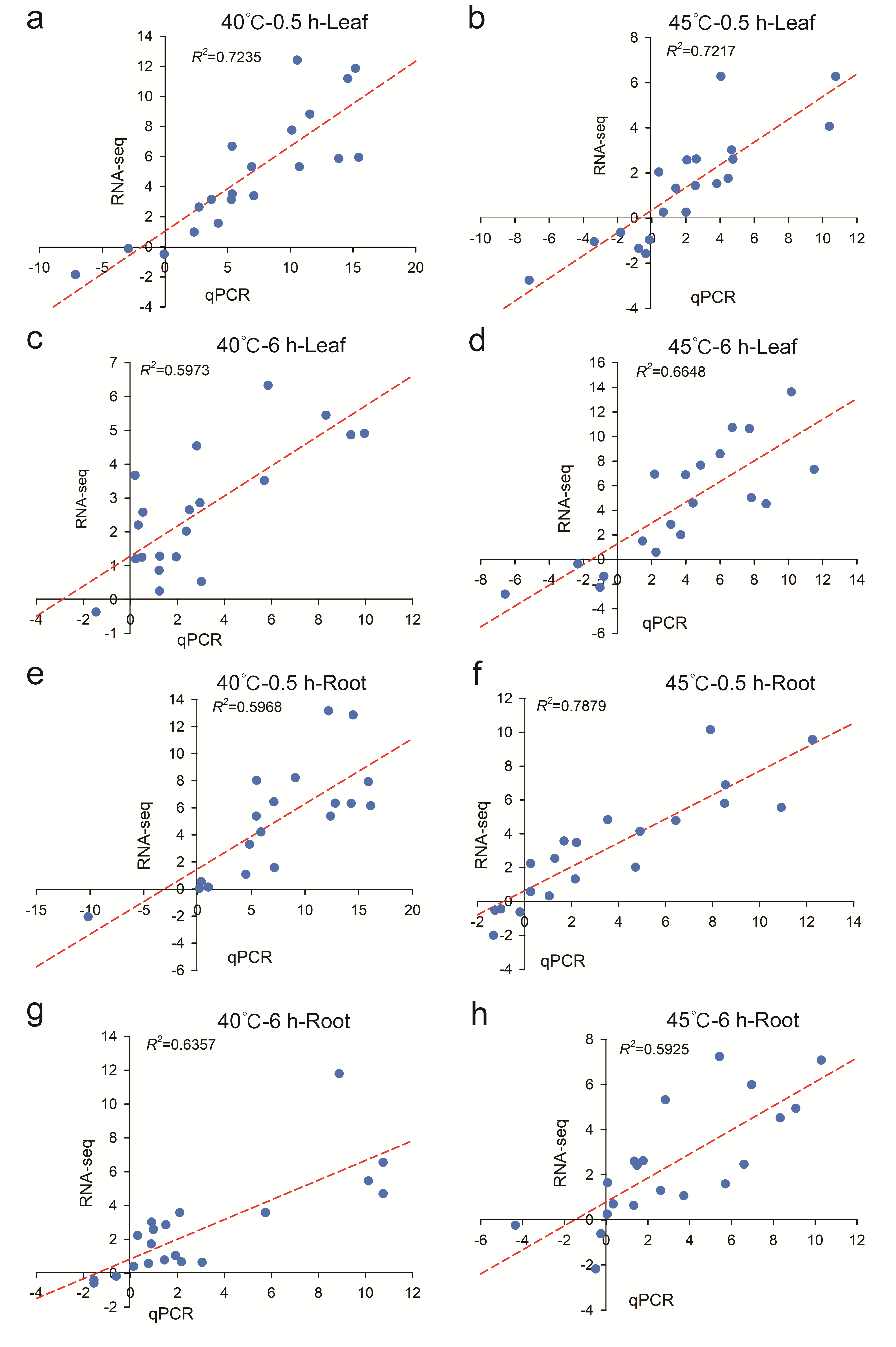


Figure S8. Correlation analysis for 20 selected DEGs between RNA-seq and RT-qPCR results in leaves (a-d) and roots (e-h) of *Z. xanthoxylum* under heat treatments.
