# Supplementary material for: Physiological and transcriptomic analyses provide insight into thermotolerance in desert plant Zygophyllum xanthoxylum
Source: BMC Plant Biol. 2023 Jan 5;23:7. doi: 10.1186/s12870-022-04024-7 (PMC9814312; doi:10.1186/s12870-022-04024-7)
Supplement: Supplementary file 2 — Additional file 2: Table S1.Overview of RNA sequencing data. Table S2. Sequencing production statistics. Table S3. Summary of sequence annotation. Table S4. DEGs related to MYBs and HSFs in roots of Z. xanthoxylum under heat treatments. Fold change equals to log2 (the RPKM value of a gene undertreatment / the RPKM value of a gene under control condition) and indicates thetranscript abundance change of each DEGs. Homologous gene and homologous species were obtained by NCBI blast according to the sequence corresponding to the gene ID. Table S5. DEGs relatedto AP2/ERFs in roots of Z. xanthoxylum identified only at 40°C for 0.5 h. Foldchange equals to log2 (40°C-0.5 h RPKM / 25°C-0.5 h RPKM) and indicates thetranscript abundance change of each DEGs. Homologous gene and homologous species were obtained by NCBI blast according to the sequence corresponding tothe gene ID. Table S6. DEGs relatedto WRKYs in roots of Z. xanthoxylum identified only at 40°C for 0.5 h. Foldchange equals to log2 (40°C-0.5 h RPKM / 25°C-0.5 h RPKM) and indicates thetranscript abundance change of each DEGs. Homologous gene and homologousspecies were obtained by NCBI blast according to the sequence corresponding tothe gene ID. Table S7. DEGs related to HSFs in roots of Z. xanthoxylum identified only at 40°C for 0.5 h. Foldchange equals to log2 (40°C-0.5 h RPKM / 25°C-0.5 h RPKM) and indicates thetranscript abundance change of each DEGs. Homologous gene and homologous species were obtained by NCBI blast according to the sequence corresponding tothe gene ID. Table S8. DEGs relatedto HSPs in roots of Z. xanthoxylum identified under heat treatments. Foldchange equals to log2 (the RPKM value of a gene under treatment / the RPKM value of a gene under control condition) and indicates the transcript abundancechange of each DEGs. Homologous gene and homologous species were obtained by NCBI blast according to the sequence corresponding to the gene ID. Table S9. DEGs related to HSPs in roots of Z. xanthoxylum [file 12870_2022_4024_MOESM2_ESM.docx]

Table S1. Overview of RNA sequencing data.

| Sample | Total Raw  Reads (M) | Total Clean  Reads (M) | Total Clean  Bases (Gb) | Clean Reads  Q20 (%) | Clean Reads  Q30 (%) | Clean Reads  Ratio (%) |
| --- | --- | --- | --- | --- | --- | --- |
| 25°C_0.5h_L1 | 47.43 | 43.64 | 6.55 | 97.16 | 89.57 | 92.01 |
| 25°C_0.5h_L2 | 47.43 | 43.99 | 6.60 | 97.15 | 89.50 | 92.74 |
| 25°C_0.5h_L3 | 47.43 | 43.96 | 6.59 | 97.40 | 89.59 | 92.68 |
| 25°C_0.5h_R1 | 47.43 | 43.91 | 6.59 | 97.02 | 89.12 | 92.58 |
| 25°C_0.5h_R2 | 47.43 | 43.77 | 6.57 | 97.43 | 89.73 | 92.29 |
| 25°C_0.5h_R3 | 47.43 | 43.44 | 6.52 | 97.57 | 90.22 | 91.59 |
| 25°C_6h_L1 | 49.19 | 45.16 | 6.77 | 97.80 | 90.94 | 91.82 |
| 25°C_6h_L2 | 47.43 | 43.40 | 6.51 | 97.66 | 90.53 | 91.50 |
| 25°C_6h_L3 | 49.19 | 44.83 | 6.72 | 97.56 | 90.26 | 91.14 |
| 25°C_6h_R1 | 47.43 | 43.40 | 6.51 | 97.56 | 90.22 | 91.51 |
| 25°C_6h_R2 | 47.43 | 43.50 | 6.52 | 97.51 | 90.03 | 91.71 |
| 25°C_6h_R3 | 49.19 | 44.78 | 6.72 | 97.56 | 90.23 | 91.04 |
| 40°C_0.5h_L1 | 47.43 | 43.39 | 6.51 | 96.93 | 88.94 | 91.48 |
| 40°C_0.5h_L2 | 47.43 | 43.50 | 6.52 | 97.50 | 89.97 | 91.71 |
| 40°C_0.5h_L3 | 47.43 | 43.36 | 6.50 | 97.02 | 89.22 | 91.42 |
| 40°C_0.5h_R1 | 47.43 | 43.56 | 6.53 | 97.06 | 89.30 | 91.84 |
| 40°C_0.5h_R2 | 47.43 | 43.92 | 6.59 | 97.04 | 89.19 | 92.60 |
| 40°C_0.5h_R3 | 47.43 | 43.79 | 6.57 | 97.16 | 89.58 | 92.32 |
| 40°C_6h_L1 | 47.43 | 43.68 | 6.55 | 97.69 | 90.57 | 92.09 |
| 40°C_6h_L2 | 47.43 | 43.19 | 6.48 | 97.52 | 90.05 | 91.07 |
| 40°C_6h_L3 | 47.43 | 43.81 | 6.57 | 97.59 | 90.20 | 92.37 |
| 40°C_6h_R1 | 49.19 | 44.89 | 6.73 | 97.60 | 90.36 | 91.27 |
| 40°C_6h_R2 | 47.43 | 43.75 | 6.56 | 97.62 | 90.35 | 92.25 |
| 40°C_6h_R3 | 47.43 | 43.75 | 6.56 | 97.69 | 90.62 | 92.25 |
| 45°C_0.5h_L1 | 47.43 | 43.66 | 6.55 | 97.05 | 89.28 | 92.05 |
| 45°C_0.5h_L2 | 49.19 | 45.02 | 6.75 | 96.99 | 89.10 | 91.53 |
| 45°C_0.5h_L3 | 47.43 | 43.43 | 6.51 | 96.92 | 88.88 | 91.56 |
| 45°C_0.5h_R1 | 47.43 | 43.29 | 6.49 | 97.03 | 89.22 | 91.28 |
| 45°C_0.5h_R2 | 50.94 | 46.75 | 7.01 | 97.48 | 89.93 | 91.78 |
| 45°C_0.5h_R3 | 47.43 | 43.35 | 6.50 | 97.60 | 90.36 | 91.41 |
| 45°C_6h_L1 | 47.43 | 42.82 | 6.42 | 97.62 | 90.44 | 90.28 |
| 45°C_6h_L2 | 46.46 | 42.75 | 6.41 | 97.71 | 90.68 | 92.02 |
| 45°C_6h_L3 | 47.43 | 43.90 | 6.58 | 97.54 | 90.09 | 92.55 |
| 45°C_6h_R1 | 45.67 | 42.40 | 6.36 | 97.44 | 89.78 | 92.83 |
| 45°C_6h_R2 | 47.43 | 43.75 | 6.56 | 97.61 | 90.28 | 92.25 |
| 45°C_6h_R3 | 47.43 | 44.03 | 6.60 | 97.57 | 90.14 | 92.83 |

Table S2. Sequencing production statistics.

| Sample | Total Number | Total Length | Mean Length | N50 | N70 | N90 | | GC (%) |
| --- | --- | --- | --- | --- | --- | --- | --- | --- |
| 25°C_0.5h_L1 | 44608 | 49939842 | 1119 | 1614 | 1135 | | 554 | 41.13 |
| 25°C_0.5h_L2 | 47229 | 55391698 | 1172 | 1676 | 1196 | | 604 | 40.97 |
| 25°C_0.5h_L3 | 43482 | 51594563 | 1186 | 1739 | 1212 | | 584 | 41.24 |
| 25°C_0.5h_R1 | 48099 | 53590931 | 1114 | 1605 | 1132 | | 555 | 41.38 |
| 25°C_0.5h_R2 | 49279 | 57673021 | 1170 | 1715 | 1193 | | 577 | 41.25 |
| 25°C_0.5h_R3 | 50585 | 55774162 | 1102 | 1638 | 1127 | | 529 | 41.17 |
| 25°C_6h_L1 | 47828 | 53838640 | 1125 | 1584 | 1135 | | 577 | 41.05 |
| 25°C_6h_L2 | 42299 | 47090063 | 1113 | 1587 | 1125 | | 560 | 41.28 |
| 25°C_6h_L3 | 48877 | 55198656 | 1129 | 1649 | 1147 | | 555 | 41.3 |
| 25°C_6h_R1 | 53005 | 58646576 | 1106 | 1645 | 1134 | | 528 | 41.17 |
| 25°C_6h_R2 | 51778 | 57363317 | 1107 | 1649 | 1136 | | 527 | 41.3 |
| 25°C_6h_R3 | 44220 | 51569900 | 1166 | 1673 | 1193 | | 590 | 41.19 |
| 40°C_0.5h_L1 | 39722 | 46057912 | 1159 | 1688 | 1173 | | 576 | 41.37 |
| 40°C_0.5h_L2 | 39673 | 46081660 | 1161 | 1689 | 1176 | | 573 | 41.26 |
| 40°C_0.5h_L3 | 39083 | 43638795 | 1116 | 1610 | 1136 | | 550 | 41.27 |
| 40°C_0.5h_R1 | 47528 | 52275868 | 1099 | 1598 | 1121 | | 545 | 41.49 |
| 40°C_0.5h_R2 | 49111 | 54288980 | 1105 | 1625 | 1123 | | 533 | 41.53 |
| 40°C_0.5h_R3 | 47130 | 52723702 | 1118 | 1635 | 1136 | | 547 | 41.69 |
| 40°C_6h_L1 | 45012 | 52537607 | 1167 | 1687 | 1189 | | 579 | 41.12 |
| 40°C_6h_L2 | 49180 | 62270399 | 1266 | 1806 | 1295 | | 654 | 40.94 |
| 40°C_6h_L3 | 51988 | 62720989 | 1206 | 1734 | 1232 | | 616 | 41.01 |
| 40°C_6h_R1 | 50188 | 57146849 | 1138 | 1669 | 1157 | | 556 | 41.31 |
| 40°C_6h_R2 | 54016 | 63157915 | 1169 | 1738 | 1192 | | 562 | 41.16 |
| 40°C_6h_R3 | 59549 | 61890900 | 1039 | 1584 | 1077 | | 472 | 40.79 |
| 45°C_0.5h_L1 | 40770 | 45996453 | 1128 | 1605 | 1135 | | 568 | 41.18 |
| 45°C_0.5h_L2 | 39425 | 46281249 | 1173 | 1693 | 1189 | | 585 | 41.3 |
| 45°C_0.5h_L3 | 40450 | 45669010 | 1129 | 1630 | 1139 | | 558 | 41.4 |
| 45°C_0.5h_R1 | 47760 | 55720703 | 1166 | 1675 | 1181 | | 589 | 41.28 |
| 45°C_0.5h_R2 | 51125 | 60808704 | 1189 | 1709 | 1210 | | 600 | 41.18 |
| 45°C_0.5h_R3 | 48413 | 55251413 | 1141 | 1662 | 1163 | | 563 | 41.36 |
| 45°C_6h_L1 | 47073 | 55019092 | 1168 | 1674 | 1191 | | 589 | 41.12 |
| 45°C_6h_L2 | 46108 | 53092842 | 1151 | 1639 | 1173 | | 582 | 41.17 |
| 45°C_6h_L3 | 51919 | 59706925 | 1150 | 1689 | 1172 | | 559 | 41.15 |
| 45°C_6h_R1 | 52068 | 61402303 | 1179 | 1733 | 1202 | | 580 | 41.2 |
| 45°C_6h_R2 | 45274 | 55137072 | 1217 | 1754 | 1250 | | 615 | 41.07 |
| 45°C_6h_R3 | 47395 | 52665375 | 1111 | 1588 | 1128 | | 555 | 41.06 |
| All-unigene | 166892 | 256035190 | 1534 | 2213 | 1595 | | 833 | 40.34 |

Table S3. Summary of sequence annotation.

| Database | Total | NR | NT | Swiss-Prot | KEGG | KOG | Pfam | GO | Overall |
| --- | --- | --- | --- | --- | --- | --- | --- | --- | --- |
| Number | 166,892 | 134,383 | 102,235 | 105,588 | 110,032 | 110,642 | 103,428 | 104,361 | 139,130 |
| Percentage | 100% | 80.52% | 61.26% | 63.27% | 65.93% | 66.30% | 61.97% | 62.53% | 83.37% |

Table S4. DEGs related to MYBs and HSFs in roots of *Z. xanthoxylum* under heat treatments. Fold change equals to log_2_ (the RPKM value of a gene under treatment / the RPKM value of a gene under control condition) and indicates the transcript abundance change of each DEGs. Homologous gene and homologous species were obtained by NCBI blast according to the sequence corresponding to the gene ID.

| Gene ID | log_2_ ratio/  40°C-0.5 h | log_2_ ratio/  45°C-0.5 h | log_2_ ratio/  40°C-6 h | log_2_ ratio/  45°C-6 h | Homologous  gene | Homologous  species |
| --- | --- | --- | --- | --- | --- | --- |
| **MYB** |  |  |  |  |  |  |
| CL958.Contig36_All | 4.90 | 7.73 | -4.98 | -4.99 | *MYB3R-1* | *Hevea brasiliensis* |
| CL11948.Contig2_All | 4.67 | 2.69 | 2.22 | 5.84 | *MYB14* | *Quercus suber* |
| Unigene63523_All | -5.10 | -5.14 | -7.65 | -3.74 | *MYB59* | *Prunus avium* |
| **HSF** |  |  |  |  |  |  |
| Unigene19442_All | 5.66 | 7.17 | 11.03 | 10.04 | *HsfA6b* | *Prunus avium* |
| Unigene19443_All | 7.43 | 9.14 | 14.33 | 12.20 | *HsfA6b* | *Prunus avium* |
| CL1594.Contig15_All | 10.17 | 5.73 | 9.81 | 7.79 | *HsfA7a* | *Ziziphus jujuba* |
| CL1594.Contig17_All | -4.84 | 4.66 | -4.27 | -3.66 | *HsfA7a* | *Ziziphus jujuba* |
| CL1594.Contig16_All | 4.68 | 8.52 | 3.71 | 2.83 | *HsfA7a* | *Ziziphus jujuba* |
| Unigene19444_All | 6.45 | 7.56 | 8.39 | 7.37 | *HsfA7a* | *Ziziphus jujuba* |
| CL1594.Contig14_All | 4.97 | 3.33 | 4.95 | 2.98 | *HsfA7a* | *Ziziphus jujuba* |
| CL1594.Contig18_All | 3.90 | 2.55 | 2.91 | 2.06 | *HsfA7a* | *Ziziphus jujuba* |
| Unigene19439_All | 10.08 | 10.81 | 8.08 | 7.60 | *HsfA7a* | *Ziziphus jujuba* |
| CL239.Contig18_All | 2.27 | 2.43 | 9.30 | 7.42 | *HsfB2b* | *Morus notabilis* |
| CL239.Contig11_All | 3.22 | 3.00 | 5.85 | 5.18 | *HsfB2b* | *Morus notabilis* |
| CL2339.Contig9_All | 2.16 | 2.70 | 3.43 | 2.21 | *HsfB4* | *Ziziphus jujuba* |

Table S5. DEGs related to AP2/ERFs in roots of *Z. xanthoxylum* identified only at 40°C for 0.5 h. Fold change equals to log_2_ (40°C-0.5 h RPKM / 25°C-0.5 h RPKM) and indicates the transcript abundance change of each DEGs. Homologous gene and homologous species were obtained by NCBI blast according to the sequence corresponding to the gene ID.

| Gene ID | log_2_ ratio/  40°C-0.5 h | Homologous  gene | Homologous  species |
| --- | --- | --- | --- |
| CL3093.Contig1_All | -7.05 | *AIL6* | *Vitis vinifera* |
| CL3093.Contig10_All | -5.21 | *AIL6* | *Vitis vinifera* |
| Unigene23646_All | 2.58 | *ABR1* | *Vitis vinifera* |
| CL3278.Contig2_All | 2.72 | *ABR1* | *Herrania umbratica* |
| CL3093.Contig5_All | -3.35 | *AIL6* | *Vitis vinifera* |
| Unigene65576_All | -5.93 | *AIL6* | *Herrania umbratica* |
| Unigene34655_All | 5.94 | *AP2* | *Trema orientale* |
| Unigene23068_All | -4.70 | *AP2-1* | *Durio zibethinus* |
| Unigene25096_All | 2.22 | *AP2-like* | *Prunus persica* |
| CL10341.Contig1_All | -0.46 | *DREB2A* | *Theobroma cacao* |
| CL9020.Contig4_All | 2.76 | *ERF014* | *Abrus precatorius* |
| Unigene13284_All | 2.74 | *ERF014* | *Populus trichocarpa* |
| CL10115.Contig5_All | 3.24 | *ERF060* | *Pyrus x bretschneideri* |
| Unigene35868_All | 3.90 | *ERF109* | *Pyrus x bretschneideri* |
| CL11297.Contig1_All | 3.98 | *ERF110* | *Ricinus communis* |
| Unigene7157_All | 2.71 | *ERF110* | *Ricinus communis* |
| CL5441.Contig3_All | 2.27 | *ERF114* | *Juglans regia* |
| CL6565.Contig2_All | 2.13 | *ERF114* | *Juglans regia* |
| CL6043.Contig10_All | 5.09 | *ERF119* | *Quercus suber* |
| CL6043.Contig7_All | 4.65 | *ERF119* | *Quercus suber* |
| Unigene48978_All | 2.46 | *ERF13* | *Nicotiana sylvestris* |
| Unigene24445_All | 4.19 | *ERF17* | *Gossypium raimondii* |
| CL8724.Contig1_All | 5.09 | *ERF1B* | *Quercus suber* |
| Unigene36358_All | -3.42 | *ERF* | *Manihot esculenta* |
| CL2493.Contig8_All | -5.18 | *ERF* | *Ziziphus jujuba* |
| CL2493.Contig6_All | 2.64 | *ERF* | *Hevea brasiliensis* |
| CL2493.Contig7_All | -3.78 | *ERF* | *Manihot esculenta* |
| CL2493.Contig2_All | -5.91 | *ERF* | *Hevea brasiliensis* |
| Unigene32286_All | -3.70 | *ERF* | *Manihot esculenta* |
| CL14118.Contig3_All | 2.20 | *RAP2-1* | *Durio zibethinus* |
| CL14118.Contig1_All | -2.28 | *RAP2-10* | *Ziziphus jujuba* |
| CL694.Contig5_All | 4.47 | *RAP2-7* | *Jatropha curcas* |
| CL13338.Contig8_All | -2.51 | *RAP2-7* | *Prunus mume* |
| CL13338.Contig15_All | -2.14 | *RAP2-7* | *Vitis vinifera* |
| CL2864.Contig5_All | 2.86 | *RAP2-7* | *Populus trichocarpa* |
| CL13338.Contig9_All | 2.69 | *RAP2-7* | *Prunus mume* |
| CL12487.Contig1_All | -3.43 | *TINY* | *Morus notabilis* |

Table S6. DEGs related to WRKYs in roots of *Z. xanthoxylum* identified only at 40°C for 0.5 h. Fold change equals to log_2_ (40°C-0.5 h RPKM / 25°C-0.5 h RPKM) and indicates the transcript abundance change of each DEGs. Homologous gene and homologous species were obtained by NCBI blast according to the sequence corresponding to the gene ID.

| Gene ID | log_2_ ratio/  40°C-0.5 h | Homologous  gene | Homologous  species |
| --- | --- | --- | --- |
| CL3929.Contig3_All | 2.20 | *WRKY 38* | *Prunus mume* |
| CL14325.Contig5_All | 2.33 | *WRKY 38* | *Manihot esculenta* |
| CL30.Contig15_All | 7.34 | *WRKY1* | *Citrus clementina* |
| Unigene11450_All | 2.52 | *WRKY15* | *Gossypium arboreum* |
| Unigene51992_All | 5.16 | *WRKY19* | *Phytophthora nicotianae* |
| CL355.Contig3_All | 3.04 | *WRKY20* | *Quercus suber* |
| CL355.Contig27_All | 5.59 | *WRKY20* | *Ziziphus jujuba* |
| CL355.Contig40_All | -3.03 | *WRKY20* | *Hevea brasiliensis* |
| CL355.Contig8_All | 2.48 | *WRKY20* | *Glycine soja* |
| CL355.Contig39_All | 3.45 | *WRKY20* | *Citrus sinensis* |
| CL355.Contig16_All | 4.79 | *WRKY20* | *Citrus sinensis* |
| CL13209.Contig10_All | 2.40 | *WRKY21* | *Larrea tridentata* |
| CL13209.Contig3_All | 3.31 | *WRKY21* | *Larrea tridentata* |
| CL13209.Contig4_All | 4.12 | *WRKY21* | *Larrea tridentata* |
| CL11193.Contig6_All | -2.63 | *WRKY23* | *Hevea brasiliensis* |
| CL2962.Contig7_All | -2.26 | *WRKY3* | *Quercus suber* |
| CL518.Contig4_All | -5.56 | *WRKY30* | *Prunus mume* |
| CL518.Contig6_All | 4.59 | *WRKY30* | *Prunus mume* |
| Unigene52382_All | 2.28 | *WRKY33* | *Theobroma cacao* |
| CL3929.Contig4_All | 3.43 | *WRKY38* | *Prunus mume* |
| CL13209.Contig6_All | 3.90 | *WRKY40* | *Quercus suber* |
| Unigene9495_All | 2.62 | *WRKY40* | *Quercus suber* |
| CL5499.Contig1_All | 3.73 | *WRKY50* | *Eucalyptus grandis* |
| CL843.Contig2_All | 2.31 | *WRKY53* | *Jatropha curcas* |
| CL9941.Contig3_All | 5.78 | *WRKY60* | *Manihot esculenta* |
| CL2851.Contig5_All | -2.07 | *WRKY61* | *Herrania umbratica* |
| Unigene23036_All | 3.25 | *WRKY64* | *Juglans regia* |
| CL9144.Contig9_All | -2.46 | *WRKY7* | *Theobroma cacao* |
| CL3178.Contig4_All | 3.60 | *WRKY70* | *Gossypium raimondii* |
| CL3178.Contig1_All | 2.04 | *WRKY70* | *Juglans regia* |
| Unigene31433_All | 2.92 | *WRKY70* | *Pyrus x bretschneideri* |
| Unigene63231_All | 2.15 | *WRKY70* | *Herrania umbratica* |
| CL7739.Contig1_All | 2.32 | *WRKY70* | *Manihot esculenta* |
| CL3929.Contig5_All | 2.66 | *WRKY70* | *Herrania umbratica* |
| Unigene5546_All | 2.16 | *WRKY70* | *Juglans regia* |
| CL3178.Contig6_All | 3.66 | *WRKY70* | *Gossypium raimondii* |
| CL1951.Contig2_All | 2.30 | *WRKY70* | *Juglans regia* |
| Unigene23576_All | 2.92 | *WRKY70* | *Juglans regia* |
| CL1951.Contig3_All | 2.46 | *WRKY70* | *Juglans regia* |
| Unigene5215_All | 5.07 | *WRKY70* | *Pyrus x bretschneideri* |
| CL7739.Contig2_All | 2.51 | *WRKY70* | *Herrania umbratica* |
| Unigene37501_All | -3.17 | *WRKY72* | *Vitis vinifera* |
| Unigene12344_All | 2.40 | *WRKY75* | *Durio zibethinus* |
| CL5450.Contig4_All | 3.08 | *WRKY9* | *Gossypium hirsutum* |

Table S7. DEGs related to HSFs in roots of *Z. xanthoxylum* identified only at 40°C for 0.5 h. Fold change equals to log_2_ (40°C-0.5 h RPKM / 25°C-0.5 h RPKM) and indicates the transcript abundance change of each DEGs. Homologous gene and homologous species were obtained by NCBI blast according to the sequence corresponding to the gene ID.

| Gene ID | log_2_ ratio/  40°C-6 h | Homologous  gene | Homologous  species |
| --- | --- | --- | --- |
| CL8607.Contig1_All | 2.80 | *HsfA2* | *Arachis duranensis* |
| CL8607.Contig2_All | 2.31 | *HsfA2* | *Arachis duranensis* |
| CL13545.Contig3_All | 2.00 | *HsfA3* | *Durio zibethinus* |
| Unigene34723_All | 4.91 | *HsfA6b* | *Ziziphus jujuba* |
| CL1594.Contig13_All | 2.32 | *HsfA6b* | *Theobroma cacao* |
| CL1594.Contig9_All | 2.06 | *HsfA7a* | *Rosa chinensis* |
| Unigene19445_All | 6.21 | *HsfA7a* | *Ziziphus jujuba* |
| CL1594.Contig7_All | 2.36 | *HsfA7a* | *Rosa chinensis* |
| CL239.Contig9_All | 3.98 | *HsfB2b* | *Morus notabilis* |
| CL239.Contig5_All | 4.23 | *HsfB2b* | *Morus notabilis* |

Table S8. DEGs related to HSPs in roots of *Z. xanthoxylum* identified under heat treatments. Fold change equals to log_2_ (the RPKM value of a gene under treatment / the RPKM value of a gene under control condition) and indicates the transcript abundance change of each DEGs. Homologous gene and homologous species were obtained by NCBI blast according to the sequence corresponding to the gene ID.

| Gene ID | log_2_ ratio/  40°C-0.5 h | log_2_ ratio/  45°C-0.5 h | log_2_ ratio/  40°C-6 h | log_2_ ratio/  45°C-6 h | Homologous  gene | Homologous  species |
| --- | --- | --- | --- | --- | --- | --- |
| **HSP100** |  |  |  |  |  |  |
| CL10111.Contig3_All | 3.72 | 6.14 | 9.59 | 8.68 | *ClpB1* | *Ziziphus jujuba* |
| CL5225.Contig12_All | 2.01 | 2.55 | 7.00 | 6.16 | *ClpB3* | *Prunus avium* |
| CL10111.Contig12_All | 10.77 | 12.25 | 7.71 | 2.61 | *ClpB1* | *Abrus precatorius* |
| CL10111.Contig2_All | 5.21 | 9.06 | 9.30 | 3.72 | *ClpB1* | *Herrania umbratica* |
| CL10111.Contig4_All | 4.03 | 7.33 | 10.04 | 4.44 | *ClpB1* | *Abrus precatorius* |
| CL10111.Contig6_All | 8.42 | 7.31 | 5.88 | 4.45 | *ClpB1* | *Abrus precatorius* |
| CL10111.Contig10_All | 2.63 | 6.53 | 13.17 | 10.32 | *ClpB1* | *Eucalyptus grandis* |
| CL10111.Contig1_All | 5.14 | 5.92 | 9.80 | 9.29 | *ClpB1* | *Ziziphus jujuba* |
| CL10111.Contig7_All | -6.66 | 6.60 | 9.05 | 6.95 | *ClpB1* | *Abrus precatorius* |
| CL10111.Contig9_All | 4.20 | 8.42 | 8.68 | 5.04 | *ClpB1* | *Ziziphus jujuba* |
| **sHSP** |  |  |  |  |  |  |
| CL3224.Contig2_All | 6.37 | 8.32 | 7.96 | 6.28 | *HSP15.7* | *Morus notabilis* |
| CL3224.Contig1_All | 3.12 | 5.99 | 8.83 | 7.90 | *HSP15.7* | *Quercus suber* |
| CL3224.Contig3_All | 8.61 | 11.80 | 9.56 | 9.53 | *HSP15.7* | *Morus notabilis* |
| CL3224.Contig4_All | 2.61 | 4.29 | 11.11 | 9.13 | *HSP15.7* | *Morus notabilis* |
| CL2261.Contig2_All | 4.19 | 6.90 | 7.87 | 6.87 | *HSP17.1* | *Vitis vinifera* |
| CL2261.Contig1_All | 5.54 | 7.42 | 8.63 | 7.33 | *HSP17.1* | *Vitis vinifera* |
| CL2261.Contig3_All | 5.34 | 7.03 | 9.13 | 7.88 | *HSP17.1* | *Vitis vinifera* |
| Unigene35445_All | 3.88 | 6.42 | 9.10 | 7.75 | *HSP17.3* | *Carica papaya* |
| CL1856.Contig2_All | 4.46 | 4.33 | 8.58 | 6.23 | *HSP17.3* | *Hevea brasiliensis* |
| Unigene54343_All | 2.91 | 5.54 | 9.15 | 8.03 | *HSP17.3* | *Gossypium raimondii* |
| CL2261.Contig6_All | 4.22 | 6.97 | 9.07 | 7.02 | *HSP17.3* | *Vitis vinifera* |
| CL1856.Contig4_All | 3.15 | 8.80 | 12.39 | 11.49 | *HSP17.3* | *Hevea brasiliensis* |
| CL2261.Contig5_All | 7.12 | 8.11 | 8.55 | 7.39 | *HSP17.3* | *Vitis vinifera* |
| Unigene21639_All | 4.46 | 5.07 | 8.46 | 7.11 | *HSP17.3* | *Carica papaya* |
| CL2261.Contig4_All | 4.28 | 8.70 | 8.16 | 7.39 | *HSP17.3* | *Vitis vinifera* |
| Unigene24714_All | 2.19 | 5.76 | 8.19 | 7.16 | *HSP17.3* | *Carica papaya* |
| CL1856.Contig1_All | 5.12 | 8.88 | 11.46 | 10.34 | *HSP17.3* | *Hevea brasiliensis* |
| CL3670.Contig4_All | 2.09 | 5.00 | 6.84 | 5.71 | *HSP17.4* | *Quercus suber* |
| CL3670.Contig3_All | 3.30 | 5.30 | 5.64 | 3.72 | *HSP17.4* | *Sesamum indicum* |
| CL3670.Contig2_All | 4.07 | 6.46 | 6.64 | 5.70 | *HSP17.4* | *Sesamum indicum* |
| CL3670.Contig1_All | 2.69 | 2.82 | 5.26 | 4.29 | *HSP17.4* | *Sesamum indicum* |
| CL13358.Contig1_All | 3.42 | 6.69 | 10.15 | 8.74 | *HSP17.6* | *Prunus avium* |
| CL13358.Contig2_All | 4.67 | 8.79 | 7.72 | 7.03 | *HSP17.6* | *Prunus avium* |
| CL9513.Contig2_All | 5.61 | 9.91 | 8.27 | 11.55 | *HSP17.8* | *Nicotiana attenuata* |
| CL8844.Contig3_All | 5.45 | 6.56 | 4.34 | 4.32 | *HSP17.9* | *Festuca arundinacea* |
| Unigene31426_All | 4.18 | 4.78 | 7.89 | 6.05 | *HSP18,1* | *Gossypium raimondii* |
| Unigene14369_All | 3.79 | 6.03 | 9.19 | 7.99 | *HSP18.1* | *Gossypium raimondii* |
| CL8000.Contig1_All | 3.35 | 3.06 | 12.11 | 12.05 | *HSP18.1* | *Populus euphratica* |
| CL8000.Contig3_All | 6.51 | 9.69 | 12.72 | 8.91 | *HSP18.1* | *Populus euphratica* |
| CL6151.Contig2_All | 4.81 | 6.42 | 7.54 | 6.13 | *HSP22* | *Medicago truncatula* |
| CL7059.Contig2_All | 3.50 | 7.28 | 10.56 | 9.05 | *HSP22* | *Ipomoea nil* |
| CL7059.Contig1_All | 4.55 | 8.01 | 9.73 | 9.88 | *HSP22* | *Ipomoea nil* |
| CL6151.Contig1_All | 5.88 | 7.46 | 6.64 | 4.83 | *HSP23.6* | *Hevea brasiliensis* |
| CL3826.Contig4_All | 5.20 | 7.07 | 9.03 | 7.80 | *HSP26.5* | *Vigna unguiculata* |
| Unigene33459_All | 4.59 | 8.56 | 8.81 | 9.91 | *HSP26.5* | *Gossypium hirsutum* |
| Unigene33461_All | 4.06 | 7.92 | 10.18 | 9.08 | *HSP26.5* | *Vigna radiata* var. *radiata* |
| Unigene33458_All | 5.28 | 6.73 | 12.26 | 9.30 | *HSP26.5* | *Gossypium raimondii* |
| CL3826.Contig2_All | 3.38 | 6.59 | 10.83 | 8.92 | *HSP26.5* | *Hevea brasiliensis* |
| **HSP70** |  |  |  |  |  |  |
| CL12652.Contig2_All | 3.30 | 8.04 | 8.54 | 5.85 | *BIP1* | *Jatropha curcas* |
| Unigene9638_All | 2.95 | 5.49 | 7.79 | 6.68 | *HSP70* | *Populus trichocarpa* |
| CL1692.Contig9_All | 3.27 | 8.40 | 8.62 | 7.79 | *HSP70.6* | *Citrus clementina* |
| CL14221.Contig7_All | 2.46 | 3.37 | 4.03 | 2.89 | *HSP70.2* | *Quercus lobata* |
| **HSP90** |  |  |  |  |  |  |
| CL1702.Contig13_All | 7.79 | 8.07 | 4.77 | -5.27 | *HSP90.1* | *Gossypium raimondii* |
| CL1702.Contig33_All | 5.72 | 7.10 | 4.94 | 5.75 | *HSP90.1* | *Populus euphratica* |
| CL12539.Contig13_All | 5.11 | 7.19 | 5.14 | 7.34 | *HSP90.2* | *Nicotiana attenuata* |
| CL1702.Contig12_All | 2.47 | 4.06 | 10.10 | 5.10 | *HSP90.5* | *Gossypium hirsutum* |
| CL1702.Contig1_All | 3.22 | 4.31 | 5.30 | 4.72 | *HSP90.5* | *Ziziphus jujuba* |
| CL1702.Contig29_All | 2.09 | 4.22 | 4.16 | 2.61 | *HSP90.5* | *Ziziphus jujuba* |
| CL1702.Contig23_All | 4.47 | 5.91 | 5.03 | -2.94 | *HSP90.5* | *Ziziphus jujuba* |
| CL1702.Contig28_All | 4.77 | 7.12 | 8.43 | 6.15 | *HSP90.5* | *Ziziphus jujuba* |
| CL12642.Contig74_All | -4.36 | -5.22 | 7.35 | 9.44 | *HSP90.6* | *Corchorus capsularis* |
| CL12642.Contig35_All | 4.59 | 4.69 | 7.57 | 7.12 | *HSP90.6* | *Vitis vinifera* |
| CL781.Contig25_All | 2.02 | 3.30 | 3.67 | 3.39 | *HSP90.7* | *Arabidopsis thaliana* |
| CL781.Contig23_All | 9.10 | 10.38 | -5.39 | -5.41 | *HSP90.7* | *Parasponia andersonii* |

Table S9. DEGs related to HSPs in roots of *Z. xanthoxylum* identified only at 40°C for 6 h. Fold change equals to log_2_ (40°C-6 h RPKM / 25°C-6 h RPKM) and indicates the transcript abundance change of each DEGs. Homologous gene and homologous species were obtained by NCBI blast according to the sequence corresponding to the gene ID.

| Gene ID | log_2_ ratio/  40°C-6 h | Homologous  gene | Homologous  species |
| --- | --- | --- | --- |
| **HSP100** |  |  |  |
| Unigene3034_All | 4.48 | *ClpB* | *Phytophthora palmivora* |
| Unigene24556_All | 2.62 | *ClpB* | *Corchorus olitorius* |
| CL5225.Contig22_All | 2.96 | *ClpB* | *Parasponia andersonii* |
| CL5225.Contig11_All | 6.21 | *ClpB* | *Parasponia andersonii* |
| Unigene24210_All | 8.34 | *ClpB* | *Parasponia andersonii* |
| Unigene22157_All | 4.19 | *ClpB* | *Parasponia andersonii* |
| CL1570.Contig12_All | 5.40 | *ClpB* | *Parasponia andersonii* |
| Unigene25730_All | 4.48 | *ClpB* | *Parasponia andersonii* |
| CL5225.Contig6_All | 5.54 | *ClpB3* | *Prunus avium* |
| CL3062.Contig2_All | 2.15 | *ClpB4* | *Glycine soja* |
| CL3062.Contig11_All | 4.82 | *ClpB4* | *Glycine soja* |
| CL3062.Contig1_All | 4.50 | *ClpB4* | *Glycine soja* |
| CL3062.Contig4_All | 3.09 | *ClpB4* | *Theobroma cacao* |
| **sHSP** |  |  |  |
| Unigene31176_All | 3.09 | *HSP17.1* | *Vitis vinifera* |
| Unigene58302_All | 4.93 | *HSP20* | *Naegleria gruberi* |
| CL2820.Contig4_All | -3.37 | *HSP17.8* | *Theobroma cacao* |
| Unigene53123_All | 5.54 | *HSP26* | *Nicotiana tabacum* |
| Unigene68699_All | 4.34 | *HSP16.9* | *Amborella trichopoda* |
| CL2820.Contig7_All | -2.84 | *HSP17.8* | *Theobroma cacao* |
| CL11472.Contig2_All | -2.70 | *HSP15.4* | *Vitis vinifera* |
| Unigene19019_All | 4.74 | *HSP18.1* | *Ricinus communis* |
| CL71.Contig2_All | 5.93 | *HSP17.7* | *oryza sativa* |
| CL10535.Contig1_All | 3.98 | *HSP17.3* | *Prosopis alba* |
| Unigene17857_All | 3.24 | *sHSP* | *Galdieria sulphuraria* |
| Unigene10959_All | 3.34 | *HSP18.2* | *Eucalyptus grandis* |
| Unigene7509_All | 5.98 | *HSP18.3* | *Tamarix hispida* |
| CL10535.Contig2_All | 4.60 | *HSP17.3* | *Gossypium raimondii* |
| Unigene31529_All | 3.80 | *HSP20* | *Actinidia chinensis* var*. chinensis* |
| Unigene63108_All | 11.64 | *HSP22* | *Theobroma cacao* |
| CL3670.Contig12_All | 7.89 | *HSP17.4* | *Quercus suber* |
| CL3670.Contig5_All | 6.80 | *HSP17.4* | *Quercus suber* |
| CL3670.Contig6_All | 6.93 | *HSP17.4* | *Sesamum indicum* |
| **HSP90** |  |  |  |
| Unigene8938_All | 2.42 | *HSP90.1* | *Populus euphratica* |
| CL1702.Contig43_All | 7.60 | *HSP90.1* | *Gossypium raimondii* |
| Unigene67418_All | 3.59 | *HSP90.1* | *Populus euphratica* |
| CL1702.Contig41_All | 2.86 | *HSP90.1* | *Populus euphratica* |
| CL1702.Contig15_All | 6.01 | *HSP90.1* | *Gossypium raimondii* |
| CL1702.Contig9_All | 3.97 | *HSP90.1* | *Gossypium raimondii* |
| CL1702.Contig36_All | 4.73 | *HSP90.1* | *Populus euphratica* |
| CL12539.Contig15_All | 2.91 | *HSP90.2* | *Nicotiana attenuata* |
| CL12539.Contig17_All | 2.34 | *HSP90.2* | *Nicotiana attenuata* |
| CL1702.Contig4_All | 2.36 | *HSP90.5* | *Ziziphus jujuba* |
| CL1702.Contig22_All | 9.74 | *HSP90.5* | *Gossypium hirsutum* |
| CL1702.Contig16_All | 7.86 | *HSP90.5* | *Ziziphus jujuba* |
| CL1702.Contig20_All | 3.77 | *HSP90.5* | *Quercus lobata* |
| CL1702.Contig45_All | 2.07 | *HSP90.5* | *Pistacia vera* |
| CL1702.Contig35_All | 5.06 | *HSP90.5* | *Ziziphus jujuba* |
| CL1702.Contig26_All | 2.21 | *HSP90.5* | *Populus trichocarpa* |
| CL1702.Contig5_All | 3.48 | *HSP90.5* | *Ziziphus jujuba* |
| Unigene55614_All | 4.48 | *HSP90.5* | *Durio zibethinus* |
| CL1702.Contig34_All | 3.45 | *HSP90.5* | *Ziziphus jujuba* |
| CL1702.Contig7_All | 4.18 | *HSP90.5* | *Ziziphus jujuba* |
| CL1702.Contig19_All | 2.77 | *HSP90.5* | *Jatropha curcas* |
| Unigene25873_All | 2.55 | *HSP90.6* | *Morus notabilis* |
| Unigene8936_All | 3.31 | *HSP90.6* | *Jatropha curcas* |
| CL12642.Contig16_All | 2.63 | *HSP90.6* | *Dendrobium catenatum* |
| CL12642.Contig75_All | 3.04 | *HSP90.6* | *Abrus precatorius* |
| CL12642.Contig45_All | 2.61 | *HSP90.6* | *Abrus precatorius* |
| Unigene49119_All | 2.69 | *HSP90.6* | *Morus notabilis* |
| Unigene8930_All | 2.54 | *HSP90.6* | *Morus notabilis* |
| Unigene19113_All | 2.24 | *HSP90.6* | *Coffea arabica* |
| Unigene2850_All | 4.93 | *HSP90.6* | *Ricinus communis* |
| CL12642.Contig18_All | 3.95 | *HSP90.6* | *Tarenaya hassleriana* |
| Unigene8932_All | 2.59 | *HSP90.6* | *Populus euphratica* |
| CL12642.Contig43_All | 2.03 | *HSP90.6* | *Olea europaea var. sylvestris* |
| CL12642.Contig69_All | 2.08 | *HSP90.6* | *Olea europaea var. sylvestris* |
| Unigene32829_All | 2.10 | *HSP90.6* | *Mucuna pruriens* |
| CL6585.Contig1_All | 5.44 | *HSP90.6* | *Jatropha curcas* |
| Unigene8917_All | 2.72 | *HSP90.6* | *Ricinus communis* |
| Unigene52181_All | 6.11 | *HSP90.6* | *Sesamum indicum* |
| CL12642.Contig37_All | 2.63 | *HSP90.6* | *Jatropha curcas* |
| CL12642.Contig41_All | 2.30 | *HSP90.6* | *Morus notabilis* |
| Unigene41865_All | 3.20 | *HSP90.6* | *Sesamum indicum* |
| CL12642.Contig8_All | 2.56 | *HSP90.6* | *Durio zibethinus* |
| Unigene29183_All | 2.21 | *HSP90.6* | *Jatropha curcas* |
| CL12642.Contig64_All | 2.34 | *HSP90.6* | *Olea europaea* var. *sylvestris* |
| CL12642.Contig21_All | 3.05 | *HSP90.6* | *Tarenaya hassleriana* |
| Unigene62589_All | 5.36 | *HSP90.6* | *Sesamum indicum* |
| Unigene8941_All | 2.55 | *HSP90.6* | *Coffea arabica* |
| Unigene50167_All | -6.19 | *HSP90.6* | *Durio zibethinus* |
| CL12642.Contig15_All | 2.23 | *HSP90.6* | *Abrus precatorius* |
| Unigene65061_All | 2.63 | *HSP90.6* | *Gossypium hirsutum* |
| CL12642.Contig44_All | 5.88 | *HSP90.6* | *Abrus precatorius* |
| CL12642.Contig27_All | 3.96 | *HSP90.6* | *Abrus precatorius* |
| Unigene49118_All | 3.26 | *HSP90.6* | *Ricinus communis* |
| CL12642.Contig70_All | 2.22 | *HSP90.6* | *Prunus avium* |
| CL12642.Contig34_All | 3.07 | *HSP90.6* | *Coffea arabica* |
| Unigene9533_All | 2.08 | *HSP90.6* | *Hevea brasiliensis* |
| CL12642.Contig38_All | 2.24 | *HSP90.6* | *Ricinus communis* |
| Unigene8916_All | 3.00 | *HSP90.6* | *Jatropha curcas* |
| CL12642.Contig12_All | 4.81 | *HSP90.6* | *Olea europaea* var*. sylvestris* |
| CL12642.Contig6_All | 2.65 | *HSP90.6* | *Ricinus communis* |

Table S10. DEGs related to PSI in leaves of *Z. xanthoxylum* identified only at 40°C for 0.5 h. Fold change equals to log_2_ (40°C-0.5 h RPKM / 25°C-0.5 h RPKM) and indicates the transcript abundance change of each DEGs. Homologous gene and homologous species were obtained by NCBI blast according to the sequence corresponding to the gene ID.

| Gene ID | log_2_ ratio/  40°C-0.5 h | Homologous gene | Homologous species |
| --- | --- | --- | --- |
| CL6683.Contig6_All | 2.39 | *PSAE1* | *Gossypium arboreum* |
| CL7285.Contig3_All | 8.16 | *PSAC* | *Castanopsis concinna* |
| Unigene22965_All | 2.19 | *PSAO* | *Juglans regia* |
| CL984.Contig8_All | 4.76 | *PSAO* | *Cephalotus follicularis* |
| Unigene52523_All | -4.90 | *PSAE* | *Gossypium raimondii* |
| Unigene48631_All | 5.90 | *PSAE* | *Cucurbita pepo* subsp*. pepo* |
| CL984.Contig17_All | -10.73 | *PSAO* | *Juglans regia* |

Table S11. The sequences used for RT-qPCR validation.

*CL1054.Contig5_All：*

ATGAGCGAACCCAACAAGAGCGGCGGCGGAGATGGTTCCGCCGCCGCGGTGCCGGCCCCGATTCCGAGTGTGAGTGGACCGCCGCCGTTCCTGAGCAAGACGTACGACATGGTCGATGACCCTTCTACAGATTCGATTGTTTCGTGGAGTCCTACGAATAACAGCTTTGTTGTGTGGAACCCGCCGGAGTTTGCGAGGGATTTGCTTCCAAAGTACTTCAAGCACAACAATTTCTCCAGCTTTGTCAGGCAATTGAATACTTATGGTTTCAGGAAGGTTGATCCAGACCGATGGGAATTTGCTAATGAGGGCTTTCTTAGGGATCAGAGGCACCTGCTTAAGAGTATAACAAGGCGAAAACCTGCTCAAGGACATGGTAATCTACAGTCTCATGGACAGAATTCCTCTGTTGGAGCATGTGTTGAGGTTGGAAAGTTTGGGCTTGAGGAAGAAGTTGAGAGGCTGAAACGAGATAAGAATGTTCTAGTGCAGGAACTTGTTAGGCTGAGGCAGCAGCAGCAGGCATCTGATACGCAGTTGCTTGCAATGGGGCAGCGTATTAAGACTATGGAGCAGCGCCAGCAGCAGATGATGTCATTTCTGGCAAAGGCTGTGCAGAGCCCTGGCTTTTTGGCTCAGTTTATGCAGCAGCAACAAAACAAGAGTAACAGGTGCAGAAGTGAAGCTAACAAGAAAAGGCGGCTGAAGCAAGACATAATTGCTGAGAGTGAGTGCCCAGCTGCTGGGCAGATTGTCAAATATCAACCTTTGAAGAATGAGGCAACTAAAGCAATGCTAAGGCAGATCATGAAAACGGATCCTTCTCCTCTACTGAGCTCATCTAAAAATGATGCTGACAGTGTCTTTATTGGGGATGGCTCATCACCATCTACTGGAATTGAGAATGGATCTTCAAGCCCTATATCTGGAGTGACTCTTCACGAAGTGCTGCCTTGTTCCGGGGGACCCTCTGTGATAACAGGGCATGCTCCAACTTCTGCCAGCTCTGACATTAATTCTTCTCCATGCAAAAAAAATTCTGAGAACGTCACAACTCATCAATTGTCTGATATCTGTAGCCTAGTTGGTTCCAAGGAGGCCCCTGCCATCTCCATCTCTCAGGCAGATATCAATCGAGAGCTTTCTCAAATATCAGATATGGCACCGGAAAATTTGGTTGATCTTCCTGTAGATGAGTATGTCGGACCTGAGCTAGGAAATGTTGGATACATGGATTCCCCTTCACTGGGAATAGAGGGGGCTATTCCGTTTGATATCGATGATATTACTCCTGATGTTGACATCGAGTCATTGCTGGATAATTCTAACTTTTGGGAAGACCTGCTTGTCCAGAGTCCAGTGCTGGAGGAGATGGAGTCAACTGCGGGGGAAGAGAATGCCAAAGAAAGCGATGTTAAACCAGTAAACAATGGGTGGGATAAAGCGCAGCATATGGATAAGTTAACAGAAAAGATGGGACTTCTCACATCAGATTCAAAAGGGGTTAATCTTTTTGACAAAAGGTAG

*CL1594.Contig16_All:*

ATGTTAATTATGATTCCTCAACTTCCAGTAAAGGAAGAGAGTACAAGGTTGAGTTCAGCTGCTCCACAGGGACCACAGCCAATGGAGGGTCTTCATGAAGGTGGCCCTCCTGCATTCCTAACGAAGACATATGACATTGTGGATGATCCAAACACAGTTGAAATAGTTTCTTGGAGCACAAGAGGTAGCAGCTTTGTTGTGTGGGATCCTCATTCTTTCTCCACTACTCTTCTTCCTAGATACTTCAAGCATAATAACTTTTCTAGCTTTGTCAGGCAGCTTAACACTTATGGTTTCAGAAAGATTGATCCTGATAAATGGGAGTTTGCTAATGAGGGATTTACCAGAGGCCAAAGGCATTTGCTGAAAAATATCAAAAGAAAAAGAGCAACTCAGTCTATTGCTCTACAGCCAGCTACGAGTCCTTGTGTCGAGCTTGGGAGTTATGAGCTAGACGAAGAAGTCAATCGTTTGAAGCGCGACAAGCAGGTTCTGCTGATGGAAGTAGTGAAGCTTAGACAGCAGCGACAGAGTACTAGAGCTTATATTCAATCAATGGAGCAAAGGCTACAGCGGACAGAAATGAAGCAGCAACAGATGATGGCTTTCTTAGCAAGGGCTATTCAGAATCCTGTCTTTGTCCAACAGCTACTTCAACAGAACGAGAAAAGGAAAGAGCTCAAAGAAGTCATAAGTAGAAAGAGAAGACGACCAATAATAGATCAAGGGCCTGGAGGAGTGGATAATGGTGGATCAAGACAATTAGGCCAAGCCGAAAACCCCATCAAAATCGAATCCATGGAGTTTGGAGATTATGATGCCAAGGTTTCCGAGCTAGAAGTACTTGCACTGGAAATGCAAGGATTCCAGAGAGTTGGAAGAGATCCTGAAGAAGAATTCAGTGAGTTAGAAACACAGGGTACTGGGGGAAGAGAACTTGACGACGGGTTCTTGGAAGAATTATTGAACGAGAGATTTGAAGAGGAATCCGACATACCGTGTGGTGAAGGAGATGGATATCAAGATATTATGTTCTTGGCAGATCAACTAGGTTAA

*CL5761.Contig5_All:*

AATGATAGGATACATCACTGTGGAGGAACGGGAAATTCTAGTGAGCAACTTGTTGCTTCCTTGTATGTACCTAGGAATGACAAGCTTGTGAAGATTGATGGAAACTTGATTTATCACTCGGTTCGGGCAAGCGAAGAAGCGGTGGCTTCTCAGGTTAAATCAGAAGCAAATAAAAGGGAAGAGACTAGTTTGGCTGTCCGTAGTGATTTAGCAGCGGCACTGGCTATTCCTGAATTTAAAAAGAATAGAGGAAAAAATCCACATGTGTATAGAAACCCGACTGAGCGTCAGAGGGCTCTTTCTTCTGGTTCCGTTGATTCCTTGAAGGACCAACCAAAATCAAGCGTTGATGGTAGGCTACAACAATGGTTCAGCGAAGGTCTTGCAGGGCCAATATTGAGTTCCGGCATGTGCACTGAAGTGTTCCAGTTTGATGTCTCTCCCACTTCTTCTCCAGGAGCCATAGTACCGGCATCTGCAATTTCTAATGTTTCTGCAGAGCGCAGGCAGAATGCTACTCACCATTATAAAGGTAGGAACAGAAGGATACTCCAGGGTATTCCAGTTTCCCTCCCTGGTTCTACTGTGAACAACACAGAAGAGCAAGTTAAAAGAGCTTCACCAAAAGATGGGTTTCAAGGAAATAAATCTGGTTCTTCTATGGTAGTTTCTGTACTTGTTGATCCCAGGGAGGTTGGTGATGGTAGTGGTGACAGTAGCATGGTGGGACCAAAGTCGCTATCGCGAATTTTTGTTGTAGTCCTTTTAGACAGTGTCAAGTATGTAACCTATTCCTGCATGCTCCCAGGAGTTGCTCCGCATCTAGTCGCAGCTTGA

*CL13209.Contig7_All:*

ATGTCTAATTTATTATCGTATATGGCACAGCTGGTGAAGGATGGATATCAATGGAGAAAATATGGCCAGAAGGTCACAAGAGATAATCCCTGTCCAAGAGCTTACTTCAAATGTTCTTTTGCTCCTAACTGCCCTGTCAAAAAAAAGGTGCAAAGAAGTATTGACGACCAATCTCTCCTAGTTGCAACTTATGAAGCCGAACACAACCATCCACCTCCTTCTCCGACAGAGGTTACTCCAGGTTTGAGTCGAGCCAATGCACCTGGTCCAGTTCCAGCCTCAGCTCCTCTATTCTCAAGTGGACCTACCCTATCTGTTGATCAGACGAAGTCTATACCATGTCAAACCGCAAAGATTGCTGTGCCTAGAGTCGAATCACCTGATTTCCAGAAGTTCTTGGTGGAGCATATGGCCTCTTCCTTGACAAAAGACCCCAATTTCACAGCAGCACTTGCGGCAGCAATTTCAGGAAGAGTCCATAGTCATTATCCTTGTGAAAATTGA

*CL239.Contig9_All:*

ATGCCAATCGACCAAGAAGCGGCAGCAATGGAGTCGCAGCGGTCACTTCCGACGCCGTTTCTGACAAAGACGTACCAGCTTGTAGAAGATCCAGCTGTGGATGATTTGATATCATGGAACGAAGATGGATCCACATTCATCGTATGGCGTCCTGCTGAATTCGCTCGTGATTTGTTGCCCAAGTACTTCAAACACAACAATTTCTCCAGTTTTGTTCGTCAACTCAACACCTACGGATTCAGGAAAGTAGTACCAGATCGATGGGAATTCGCGAACGATTGTTTCAAGAGAGGAGAGAAGAGTCTTCTACGCGACATTCAACGACGGAAAGTATCACCTTCTCCTACTACGACACCGTCCGTGGCGGCTGCAATCCCCGCGGTGGCAGTCACTGTGGCAGCGGTGCCGGATGTGGGGGCAGTGGTGTCTCCGTCTAACTCAGGGGAAGAGCAGGTGATCTCGTCAAACTCATCTCCGTCGATGGCGGCTCTCCACCGTGGAACGAGTTGCACCACCGCGCCTGAGCTGATAGAGGAGAACAAAAAGCTGAGGAAGGAGAACGTGCAGTTGAATCAAGAGGTTAGTCAACTGAGAGGGTTGTGTAATAACATCATGGCTTTGATGAACAATTTCGCGCCTTGTTCTTCTTCGGATTCTGCGGTGGAAGAGAAGACTCTGGAGTTGATGGGAGTGAGGCAGGTTCCGGCGGAGGAAAGCGGTGGCGCCGCCGTGAGTGGTGGAAAGGAGGAGATTAATACGCAGAAGTTGTTTGGAATATCGATTGGGGTGAAGCGCGTGAGGGATGAGGCGGTGGATGGCATAGCAGCTGCCAATCAGCGGATGACAGACGACGAAAAGGATGGTGTTGTTGGTGTTGGCTCGGATATGAAAGCGGAGCCGTCCGATGTTAGTGGGGAGAATCAGGACCGTCCATGGTTGAAACTTGGAAAATCATGA

*Unigene30694_All:*

ATGTTGGCAAATAACGTTGTTGGAAGGAAAAGGAAGTCTAGGAGTAGACGAGTTGGATCGAAATCAGTGGCTGAGACGCTTGCTAAATGGAAAGAATACAATGCTCGGCTCGAATCTTCTACGGACGCGAGCAAGCCTACGCGGAAGGTCCCTGCTAAAGGATCGAAGAAAGGGTGTATGAAGGGGAAAGGAGGGCCTGACAATTCTCGGTGTAATTACAGAGGTGTTAGGCAGAGGACATGGGGAAAGTGGGTTGCTGAAATTAGGGAACCCCACAGAGGAAACAGGCTATGGCTTGGCACTTTCCCAACTGCTTACGAGGCTGCTCTTGCTTACGATGAAGCTGCAAGGGCCATGTATGGTCCATGTGCTCGACTTAACATGCCAAATATTAATTCCTCTAGGGTGGACTCGAAGGATGAATCTTTAGATGCAGGATGCTCGTCTTGCTCCTCATGTGTAGAGATGCCTGCAGGTTCTGATTTGACTACGGCTTCGTTGATATCGAAACAATCTGATGCTTCTACTGCTGATCCCAGTGAAACATGCGCTTCGTCCAATATGAAACGCGGGGATGATACTGACGCTGCTACTCTATCCAATATGAAACGTGGGGATGATACTGATGCCAGTACTCCCATTAGTATGGTTAAGTCAGAGGTTCAGGATGAGTCTTCTGATGCAACAGATCACGACATCTGTGGTGCACCGAGGATGAGCGTGGAGCCGAAGGATAATTCCAAGAAGGAAGAAAGGAAAGACGAGCTGCCGTTGGATGTTGATGATTACAAATGGATGGAGGGACTTGATATTGGGCAGGATTGTCTGGGAAGCTATACCATCGATGAAATGTTTGATATGGATGAGCTCTTGGGCGATATAGACAACAGCCCCCTTGGAGGTCCCAATTTTTTGGATGACTTAGACGGGTTGGGAGGAGGTACATTAGGAAATCCTAGCGATGAGCCAGAGAAACAGGGTGAGAAAGCTGCTGACTTCTCATATCAGCTTCAGAACCCTGATGCCAAACTACTCGGAAGTCTGCAACATATGGAGCAACTGCCTACAGGCATGGACTATGGTTTCGATTTTCTAAAGTCGGATACGCCCGAGCAGAATGACATGAAGCTCGAGGATCCAAGCTTCTTGAATTTTGATATATGTGATTTTGAGATATGA

*CL9799.Contig1_All:*

ATGCCAACAAGGTCAACAGGAACCATAACCCAAGACTGGCAGCCAGTGGTACTCCACAAATCCAAGACCAGAGCACAGGACTTACGCAACCCAAAGGCTGTCAACAGTGCACTTCGGTCAGGAGCACCGGTCGAGACAATCAAGAAATCCGACGGCGGGTCGAACAAGAAGACATCAGGAGGGCCAGTCATTAACGCGAGGAAACTCGACGAGGCTGCTGAGCCAGCTGCGTTGGACAAGGTTTCTGTAGACGTGAGGCAGGCTATACAGAAGGCAAGGCTTGAGAAGAAGATGAGCCAAGCTGAGTTGGCTAAGCAGATAAACGAGCAGGTTAAGGTTGTCCAAGAGTATGAGAATGGTAAAGCTGTGCCTAACCAGGCTGTGTTGGCTAAGATGGAGAGAGTTTTGGGTGTTAAGCTTAGGGGAAAGAAGTGA

*CL1702.Contig18_All:*

ATGAGGAGTCCAAAAGATGTCGAGAAAGAGGAGTACCAAAATTTCTACAAGAAGACCTTTAATGAATTCTTAGACCCATTGGCATACGCTCACTTTACAACCGAGGGTGAGGTGGAATTTAGAAGTGTTTTATATGTCCCCGGAATGGGACCTCTTAACAATGAGGAAGTTGCCAATCCAAAAACAAAGAACATACGTTTATACGTAAAGCGTGTATTTATATCAGATGATTTTGATGGCGAGCTGTTTCCACGATACTTGAGTTTTGTGAAAGGTGTTGTGGATTCAGATGACCTTCCTCTAAATGTGTCTCGTGAGATCCTTCAAGAAAGCCGTATTGTGAGAATAATGAGGAAAAGACTTGTTAGGAAGACATTTGACATGATCCAAGACATTGCAGACAGTGAGAACAAAGAGGACTACAAAAAGTTCTGGGAGAACTTCGGCAGGTTTATCAAGTTAGGATGCATTGAAGACTCTGGAAACCACAAGCGCATATCACCACTGTTGCGGTTTTATTCCTCCAAAAATGAGGAAGAATTGACTAGCTTAGATGATTATGTTGAGAACATGCAGGAGAACCAGAAGGCAATCTATTACTTGGCCACAGACAGCTTGAAAAGTGCAAAGACAGCCCCATTTTTGGAGAAGCTGGTTCAGAAAGATATTGAGGTTCTTTATTTAGTTGAGCCGATTGACGAAATTGCTATTCAAAACTTACAAACCTACAAGGAAAAGAAGTTTGTTGATATCACAAAGGAAGATTTAGAGCTTGGTGATGAGGACAAGGCCGAAGATGAGGAAACAAAACAAGAATTCAATCTTCTGTGTGATTGGGTTAAACAGCAATTAGGTGATAAGGTGGCCAAGGTGCAGGTCTCCAAGCGTCTAAGTTCATCCCCATGTGTTCTTGTTTCTGGAAAGTTTGGATGGTCCGCTAACATGGAAAGGCTGATGAAGGCCCAAGCTCTTGGGGACACCTCAAGTTTGGAGTTTATGAGGGGTAGAAGGATTCTAGAGATCAATCCAGATCATCCCATCGTCAAAGATCTAAATGCTGCATGTAAGAACGCACCTGAAAGCTCGGAGGCCAAGAGGGCTGTTGATCTCTTATTTGACACAGCATTGATCTCCAGTGGATTCACGCCGGATAGTCCTGCTGAGTTGGGAAACAAGATATATGAAATGATGGCCCTTGCGCTTGGAGGAAGATGGGGCAGATCTGAAGCAGAAGATGCAACTGAAGAGAATGCTGCTGAATCTAACACAAGCTCTACCGAAGTCTCCCAGCCCGAAGTAGTGGAACCATCCGAAGTGAGGACGGAAAGCGATCCTTGGCAAGAATAG

*CL2232.Contig10_All:*

ATGAGCGTGGTGGGTTTTGATTTCGGTAATGAGACCTGTGTGGTTGCCGTAGCGAGGCAGAGAGGGATCGACGTTGTGCTCAATGACGAATCCAACCGTGAGACTCCCACTATTGTTTGCTTCGGCGAGAAGCAACGGTTCCTTGGTACTGCCGGAGCTGCTTCGATTACTATGAATCCCAAAAACTCGGTTTCTCAGATTAAGAGGTTGATTGGGAGGAAATTCAGTGATCCGGAGCTGCAGAGAGATCTGAGGTCCTTGCCTTTTCAGGTGTCGGAAGGGCCGGATGGGTATCCTTTGATCCATGCTCGGTATCTTGGGGAAGTGAGATCTTTTACTCCTACTCAGGTCATGGGGATGATGTTCTCTAATCTTAAAAATATAGCTGAGAAGAATCTCAATGCCGCGGTTGTGGACTGCTGTATTGGTGTTCCTGTTTATTTTACTGATTTGCAAAGAAGGGCTGTCATGGATGCAGCTACGATTGCGGGTTTGCATCCGTTGCGTTTGATCCATGAGACCACTGCGACAGCATTGGCTTATGGAATTTACAAAACGGACTTGCCTGAGAATGACCAGTTAAATGTTGCTTTTGTTGATGTTGGACATGCCAGCATGCAAGTTTGTATTGCTGGTTTTAAGAAAGGGCAGTTAAAGATATTGGCTCACTCCTATGATAGTTCTTTGGGTGGTAGAGATTTCGATGAAGTTTTGTTTCACCATTTTGCAGCAAAGTTTAAGGATGAGTATAAGATTGATGTTTTTCAGAATGCAAGGGCTTGCCTCAGGCTTCGTACTGCCTGTGCAAAATTGAAGAAGGTTCTTAGTGCTAACCCCGAGGCACCTCTTAACATAGAATGCTTAATGGATGAGAAGGATGTTAGAGGCTTCATTAAAAGGGAGGAGTTCGAGAAAATTAGTCTTCCAATACTTGAACGTGTGAAGGGGCCTTTGGAGAAGGCACTGAAGGATTCTGATCTTTCAGTTGAGAATATTCATTTTGTTGAAATTGTTGGATCTGGATCTCGGGTTCCAGCCATTTTAAAGATATTGACAGAGTTCTTCGGTAAAGAGCCCAGGCGTACAATGAATGCTAGTGAATGCGTTTCTAGAGGCTGTGCATTGCAATGTGCTATTCTTAGTCCCACGTTTAAAGTGCGGGACTTCCAGGTGAATGAGCACTTCCCATTCTCCATTTCTTTGTCATGGAAAGGTTCTGCTCCAGAGGCTCAAAATGGAGCAGCTGAGAGCCAGCAGAGCATTGTGGTTTTCCCCAAGGGTAACCCAATTCCTAGTGTGAAGGCTCTCACATTCTACAGATCAAGCACGTTTGCTATTGATGTACAATATACGGACCCAAATGAAGTGCAGGCACCAGCAAAAATAAGTACATACACGATTGGTCCATTTCATCCTCCTAATCGTGAAAGGGCAAAACTGAAGGTGAAAGCTCGCTTGAATCTGCATGGGATTGTGGCTATCGAGTCCGCAACACTCATTGAAGAAGAAGAAGTTGAAGTTCCAGTATCCAAAGAACCGGAAAAAGAAGCCAACAAGATGGATACTGATGAAGCTACTTTTGAAACTGCGCAACCTCCTGCAAACGAGTCTGATGTAAACATGCAAGATGCCAAGGGTCCTATTGATGCCCCTGGTTCTAATAATTGTGTCCCTGAGGCAGGAGACAAACCGGTTCAAATGGAGACTGACACAAAGGTGGAGGCTCCTAAGAAAAAGGTAAAGAAGACAAATGTTCCTGTGACGGAGATAGTCTACGGTGGAATGGTGCAAGCAGATGTGCAGAAGGCCGTGGAGAAAGAGTTCGAGATGGCTTTACAGGATAGAGTGATGGAAGAAACCAAAGACAAGAAAAATGCCGTGGAGGCTTATGTCTATGACATGAGAAATAAGGTGGACAGTTCTTAA

*Unigene9635_All:*

ATGGCCAAGACTGAAGGGAAGGCTGTTGGTATTGATCTTGGCACAACCTACAGCTGCGTAGGAGTGTGGCAAAATGACAGGGTTGAGATCATTGCCAATGACCAAGGTAACAGGACAACTCCATCCTATGTTGCCTTCACTGACACTGAACGGTTGATTGGCGATGCAGCCAAGAACCAAGTTGCCATGAATCCTCGGAATACTGTTTTCGACGCAAAACGACTCATCGGCAGAAGGTTTTCCGACCCTCCTGTCCAGAGTGACATGAGGCATTGGCCTTTCAAGGTTGTTCCAGGCCCTGCTGATAAGCCTATGATTGCTGTGACTTATAAGGGTGAAGAGAAGCAGTTTGCTGCTGAGGAGATTTCTTCAATGGTGTTGACAAAGATGAAAGAGATTGCTGAGGCTTATCTTGGCCAGACTGTCAAGAATGCTGTGATCACTGTGCCTGCATACTTTAATGACTCTCAAAGACAGGCTACTAAGGATGCTGGGGCTATTGCTGGACTCAACGTGATGAGAATTATCAATGAGCCTACTGCTGCTGCCATTGCTTATGGTCTTGACAAGAAGAAGGCTTCGAGCAGAGGTGAAAAGAATGTGCTGATTTTCGATCTCGGCGGCGGGACTTTCGACGTTTCGTTGCTGACAATTGAAGAAGGGATCTTCGAGGTTAAGGCTACTGCTGGTGATACTCATCTTGGAGGTGAGGATTTTGATAACAGGCTTGTGAACCATTTTGTGGCAGAGTTCAAGAGGAAGAGTAAAAAGGATATTAGTAACAATGCTAGAGCATTGAGGAGGCTGAGGACTGCTTGCGAGAGAGCAAAGAGGACATTGTCATCCACCACTCAGACAACAATTGAGATTGATTCTCTTTATGAAGGTATCGATTTCTATTCTACTATTACTCGAGCTAGGTTCGAGGAGTTGAACATGGATTTGTTTAGGAAGTGTATGGAACCTGTTGAAAAATGTCTTCGGGACTCTAAGATTGATAAGAGTCAGGTTGATGATGTTGTTCTCGTCGGCGGGTCGACGAGGATTCCAAAAGTTCAGCAGCTCTTGCAAGATTTCTTCAATGGGAAGGAATTGTGCAAGAGCATTAACCCTGACGAGGCTGTTGCCTATGGAGCAGCTGTCCAGGCTGCAATTCTTAGCGGAGAAGGAAATGAGAAGGTTCAGGACTTGCTTTTGCTCGATGTTACGCCTCTGAGTCTCGGTATTGAGACTGCTGGAGGTGTAATGACAGTCCTGATTCCAAGAAACACAACAATTCCGACGAAGAAAGAGCAGATTTTCTCGACTTATTCGGACAACCAACCTGGAGTGCTAATTCAGGTGTATGAAGGTGAGAGAGCCAGAACCAGGGACAACAATCTTCTTGGGAAGTTTGAGCTCACAGGCATTCCACCTGCTCCTAGAGGTGTCCCTCAGATCAATGTCTGCTTTGACATCGATGCAAATGGCATACTGAATGTCTCCGCCGAGGACAAGACTGCCGGAGTGAAGAACAAGATAACAATCACCAATGACAAAGGGAGACTAAGCAAGGAGGAAATAGAGAGAATGGTGCAAGAGGCAGAGAAGTACAAGGCTGAGGATGAGGAGGTGAAGAAAAAGGTTGAAGCCAAGAACTCTCTGGAGAATTATGCTTACAACATGAGGAACACTATCAAGGACGAGAAGTTCGCTGGAAAGCTGAACCCAGCAGACAAGCAGAAGATTGAGAAAGCAATTGATGAGGCAATGGAATGGCTTGAGAAAAACCAGTTAGCTGAGGTTGATGAATTCCAAGACAAACAGAAAGAATTGGAGGGCCTTTGCAACCCCATTATTGCTAAAGTGTACCAGGGGGCAGGAGGAGATGTGCCTATGGGTGGTGGAGCTGACATGCCAAGTGGTGGCTCTGGTGCTGCTGGTCCTAAGATTGAAGAGGTGGATTAA

*Unigene33461_All:*

ATTTGTCCGTATTTATTTTCATCAAACACTGACAGCAGCAAAAAAAATCCTGACTTTCAAGTGTCTGCTCTTTTACCCAGTTTCTTTACAAGATATTTGGCAATGGCTTATGCTCGTTTGGCTTTGAAGAATCTCCAGCAAAGGGTATGCTCTTCTCCTGTTGCGAGCAACAACATCCGAGAAAGAGCTTTTGCTAGTGTAGAAAGGCAGAGGTGGGGAAGTGAGTATTCAGGGAGGTTTTTGGCTACTGCGGCTGGTGATACAAAGGCCCCCGAAGAGAACAATGAAGGTGGCAAAGAAGTTGCTGTCACCAAAGGCAGGAAGAAGTCCTCTAGACTCAATAGACTATTACCAAGGTGGCCAAGGAGGAGAGGCCTTTGGAGGGACAGTGACAGAGACTTTTCTCCTGCTCTTCCAGAGCTTTTTCCATCAGGGCTTGGGAATGCACTGATGCAAGCAACAGAGAACATAAACAGGCTGTTTGATAACATGAGCTTGACCACCCCATTTCAAATGTCTGGACGTATGAAGGAGCAGGATGAGTGTTACAAACTGAAGTTTGACATGCCAGGGATTCCCAAAGATGAAGTGAAGATAACAGTTGATGATGGAGTTTTGAGAATCAAAGGAGAGCACAAACAAGAGGATGAAGAAGAGTCTGATGATGAGTACTGGTCTTCAAGGAGTTATGGCTACTATGACACAAGCCTTATGTTGCCTGATGATGCCAAGGTTAATGATATTAAGGCTGAGTTGAAGCATGGTGTGCTTCATATCACAATTCCTAGAACTGAGAAGTCCAAGAAAGATGTTAAAGAAGTTCAAGTGAATTGA

*CL10111.Contig12_All:*

ATGAATCCTGACAAGTTCACACACAAGACTAATGAGGCCCTTGCTTCGGCTCACGAGTTGGCCATCAATGCGGGGCATGCTCAGTTTACACCTCTGCATTTGGCTGTTGCTCTGATATCTGATCCCACTGGAATCTTTAGCCAAGCAATCCAAAGTGCTGGAGGCGAGGATGCTGCGAAGTCGGCGGATAGGGTGTTTAATCAAGCTTTGAAGAAACTGCCCTCGCAGTCTCCTCCTCCTGACGAAATCCCAGCTAGTACAACTCTGATTAAGGTGATTCGGAGGGCTCAAGCGGCACAGAAAGCTCGCGGGGACACGCATTTGGCTGTGGATCAGTTGATCATTGGTCTTCTTGAAGATTCACAAGTTGGTGACTTATTGAAAGAATCTGGGGTTGCGTCTGCTCGGGTGAAGTCTGAGGTAGAGAAACTGCGAGGGAAGGATGGGAAGAAAGTTGAAAGTGCTTCCGGGGATACTACATTCCAGGCTTTGAAAACCTACGGGCGTGACCTTGTTGAACAGGCTGGGAAGCTGGATCCCGTGATTGGTCGGGATGATGAAATCAGGAGGGTAATTAGGATTCTTTCGAGGAGAACCAAGAATAATCCTGTCCTTATCGGAGAGCCTGGCGTGGGTAAAACTGCAGTTGTAGAAGGATTAGCTCAGCGAATTGTCCGAGGTGATGTCCCTAGTAACCTTTCTGATGTGAGGGTTATAGCATTGGATATGGGTGCTCTTGTTGCTGGGGCAAAATATAGAGGAGAATTCGAAGAAAGGCTTAAGGCTGTTTTGAAAGAAGTGGAAGAAGCTGAAGGAAAAGTGATCTTATTCATTGACGAAATCCATCTTGTACTTGGGGCGGGTCGCACGGAGGGGTCAATGGATGCGGCAAATCTGTTTAAGCCGATGCTTGCTAGGGGGCAGCTCCGATGCATTGGTGCGACTACACTGGAGGAGTACAGGAAATATGTTGAAAAAGATGCAGCATTTGAAAGAAGATTCCAGCAAGTTTACGTGGCCGAGCCTAGTGTAGCTGATACAGTTAGTATTCTTCGTGGGCTGAAAGAGAGATATGAAGGTCACCATGGTGTTAGAATTCAAGATCGAGCTCTCGTTGTTGCTGCACAGCTTTCAAGCCGATACATCACTGGTCGCCATCTACCTGACAAGGCTATAGATTTGGTTGATGAAGCATGTGCAAATGTGCGAGTTCAACTCGACAGTCAGCCTGAGGAAATTGATAATTTGGAGAGGAAGAGAATGCAGTTAGAGATTGAACTTCATGCCCTCGAAAAGGAGAAAGACAAGGCTAGTAAAGCCCGTTTTGTTGAAGTTCGGAAGGAGCTTGATGATCTTAGGGATAAGCTTCAGCCTCTTATGATGAAGTACAGAAAGGAGAAAGAAAGAATTGATGAGATTAGACGATTGAAGCAGAAAAGGGAAGAGCTCTTAATTTCTTTACAAGAAGCTGAAAGAAGATATGATCTAGCCAGAGCAGCTGACTTGAGATATGGAGCAATCCAGGAAGTTGAATCTGCCATACAACAACTTGAAGGAAGCACCGACGTCAATTTAATGTTAACCGAGGCTGTTGGACCGGAACAAATTGCAGAGGTGGTGAGTCGCTGGACAGGAATTCCGGTGACTAGGCTAGGACAGAATGACAAGGAAAGGTTAATGGACTTGGGTGAAAGACTGCACCAAAGAGTTGTAGGACAAGACATTGCAGTTGATGCTGTTTCAGAAGCAGTATTGAGGTCAAGGGCTGGATTGGGGAGACCCCAACAGCCTACTGGGTCATTCCTGTTCCTTGGTCCAACTGGTGTTGGTAAAACTGAGCTTGCAAAAGCTCTTGCTGAACAACTTTTCGACGATGAGAATCTACTCGTTAGGATCGATATGTCCGAGTATATGGAACAGCACTCAGTAGCGCGATTGATTGGTGCTCCACCTGGGTATGTCGGACATGAGGAGGGTGGTCAATTAACTGAGGCTGTAAGGCGTAGGCCTTACAGCGTTATTCTATTTGATGAAGTGGAGAAAGCACACATTTCGGTTTTCAACACTCTACTTCAGGTCCTGGATGACGGAAGGCTAACAGATGGCCAGGGCAGGACTGTAGATTTCAGGAACACAGTTATTATTATGACTTCCAATCTCGGAGCAGAGCTTCTCCTGCAAGGGCTCACAGGGAAGGTCTCGATGCAAGTTGCCCGTGATCGTGTTCTGCAAGAAGTGAGAAGGCACTTCAGGCCCGAGTTGTTAAACAGGCTTGATGAAATTGTGATATTCGATCCGCTCTCACATGAACAGCTGAGAAAGGTAGCAAGACTACAAATGAAAGATGTTGCTGCTCGCCTTGCAGAGAGAGGTATTGCTCTGGCTGTAACGGATGCGGCACTGGACTTTGTGCTGGCAGAGAGCTATGATCCGGTAAGTAACTTTAAAGTTACCAAAAATTGTTCACACACGTTCAAATTTTCGGAGAAGTACTCAAATTATGTTATGATCCGGTTCAGGTTTATGGTGCTCGGCCTATCAGAAGATGGCTCGAGAAGAAGGTTGTGA

*CL12279.Contig14_All:*

ATGTTTGGAAGAGCAGCACCAAAGAAGAGCAACAACACAAAGTACTATGAAATTCTTGGAGTGTCAAAGAATGCAACACAAGAAGACTTGAAAAAAGCTTACAAGAAAGCTGCTATTAAAAACCATCCTGACAAAGGCGGTGATCCTGAAAAGTTCAAAGAGTTGGCTCAGGCGTATGAAGTTCTGAGTGATCCTGAAAAACGAGAGATATATGATCAGTATGGAGAGGATGCTCTTAAGGAAGGCATGGGTGGTGGTGCAGCTGCTCATGACCCTTTTGATATCTTCTCCTCTTTCTTTGGCGGTAATCCTTTTGGAGGTGGGAGCAGTAGGGGAGGTAGAAGGCAGAGGAGGGGAGAGGACGTGGTGCATCCTCTAAAGGTGTCGTTAGAGGATCTTTACTTGGGGACTAGGAAGAAACTCTCTCTTTCTCGGAATAAGATATGTTCAAAGTGTAATGGTAAAGGGTCTAAATCCGGGGCATCGATGAAATGTCCTGGCTGTCAAGGTACGGGTATGAAGGTTACAGTTAGGCATTTGGGACCTTCGATGATTCAGCAGATGCAGCATGCTTGTAATGAATGCAAAGGTACTGGAGAGTCCATTAGTGATAAGGATCGGTGCTCGCTGTGTAAAGGTGAGAAGGTTGTTCCAGAGAAGAAGGTGCTTGAAGTTATTGTTGAGAAGGGTATGCAGAATGGGCAGAAAATTACATTCCCTGGTGAAGCAGACGAAGCTGTAAGTTCTTCTTGTAGCTGA

*CL1088.Contig11_All:*

TGTAAGGCTTTGTTTTTGTTTGTTGTGTTGTTGCTTGTTGAAGTGATGGGAAAGTCTTACCCGAGTGTGAGTGAGGATTACCAGAAGGCTGTTGAGAAGGCTAAGAAGAAGCTCAGAGGTCTCATTGCTGAGAAAAACATCGCTCCTATTATTCTTCGCCTCGCATGGCACTCTGCTGGTACTTTCGATGTGCAGAGCAAGACTGGAGGTCCATTCGGAACCATTAAGCACGCTGATGAGCTGAAACATGGTGCTAACAATGGTCTCGACATTGCTGTTAGGCTAGTGGAGCCGATCAAGGAGCAGTTTCCTAACATCTCTTATGCTGACTTCGTACAGTTGGCTGGAGTTGTTGCTGTTGAGGTCACCGGTGGGCCTGAAGTCCCCTTCCACCCAGGAAGAGAGGACAAACCCCAGCCACCACCTGAGGGTCGTCTGCCTGACGCTACTAAGGGTTCTGACCATCTGAGGGATGTATTTGGAAAACAAATGGGCCTCAGCGACCAGGACATTGTTGCTCTCTCTGGTGGCCACACCTTGGGAAGGTGCCACAAGGAGAGGTCTGGATTTGAGGGACCCTGGACCAGCAACCCTCTCATCTTTGACAATTCCTACTTCAAGGAGCTTTTAAGTGGCGAGAAGGAAGGACTTCTTCAATTGCCCTCCGACAAGGCTCTTCTTTCTGATCCAGTCTTCCGCCCCCTTGTTGAGAAATATGCTGCTGACGAAGATGCTTTCTTTGAGGACTACAAGGAAGCTCATTTGAAGCTTTCCGAGCTTGGGTTTGCTGAGGCTTGA

*Unigene18687_All:*

CGAAATCTAACTTGGAGAGAAATGACTATCATCACAGCCAACACTGCTGCCTCATCTATCGTTCGCCCAATTCTTTCCCAGAAAAGATTTTCAAGAGTTCCTTCAACTGTCCTTGGGCTGCCAGCAGCTATGTCAAAGAAGGGAAGAGTGAGTTGCTCCGTAAAGGAAAGGGCATCAGTTGAGAAGGACTCAACCGTGGGCATGGGTGCATCTTTGATGGCAGCGATTATGGCTGCGAGCATGTCAAGTCCAGCAGCCTTGGCTCTTGTGGATGAGAGAATGAGCACTGAAGGAACAGGATTGCCATTTGGGTTGAGCAACAACCTTCTTGGTTGGATCCTGTTTGGTGTGTTTGGTCTGATCTGGTCTCTTTACTTTGTCTACGTTTCCGGACTTGAAGAGGACGAGGAGTCTGGATTGTCCCTGTGA

*CL411.Contig4_All:*

CTTATCTTACGTTGTCTGTCGGTCGCACAAAGCCGTATTTTCAAAATGCATTTATGCCGCACACTACCATCATCTTTATTCGCCTTTCCTTGCCTTCCAAACTACCAAGTCAGTGGCGGAAATGATGTTGTGGTTCCTAAACACAAGAGTGTCTACAGCATTAGAAGACTTGTGGCTAAGGCCTATTCTGCTGATAAAATGGCTTCAAATGATAGTGATGTACCCTTTCCGACAGATTATACAATGCTTTTGGAGCAAGCCAAAGCAGCTACTGAGTTGGCTCTGAAGGACAACAAACAAGTACTGGAAATTGAGTTTCCAACCTCGGGACTGAGATCCGTACCAGGTGATGGTGAAGGAGGGAATGAAATGACTGAAAGTATGCAATTTATTCGTGAATATTGTGACCGGCTGATAAATCCAGAGAAAGTATCACGAACAAGGATATTTTTTCCTGAAGCTAGTGAAGTTAGCTTTGCAAGGCAATCAGTCTTTGAGGGGGCTTCCTTTAAGTTGGACTACCTGACAAAGCCATCATTTTTCGAGGATTTTGGTTTTGGCACAAAGATAAGAATGGCAGACCGTGTCAAGCCAGAAGATGAACTGTTCCTTATTGCTTATCCTTATTTTAATGTTAATGAAATGCTTGTTGTGGAGGAACTTTACAAAGAAGCTGTAGTGGGAACTTCCCGGAAACTTATCATATTCAACGGAGAACTTGATCGCATAAGATCTGGATATTATCCATCATTCTTCTATCCAAAGCTGGCAGCACTGTTGAAGACTCTGTTCCCCGCAATGGAAACTGTATATTACATTCACAATTTCAAGGGAAGCACTGGTGGAACCCTTTTTAGGTGCTACCCAGGCCACTGGAAAGTCCTCAGAAAAGTGAGGAACGAGTACATTTGTTTGCATCAGCAGGAAGATATGCCATCACTTAAAGAAGTTGCTTTGAACATTCTTCGATGA

*Unigene40490_All:*

CGAAATCTAACTTGTAGAGAAATGACAATCATCACAGCCAACACTGCCGCCTCATCTATCGTTCGCCCAATTCTTTCCCAGAAAAGATTTTCAAGAGTTCCTTCAACTGTCCTTGGGCTGCCAGCAGCTATGTCAAAGAAGGGAAGAGTGAGTTGCTCCGTAAAGGAAAGGGCATCAGTTGAGAAGGACTCAACCGTGGGCATGGGTGCATCTTTGATGGCAGCGATTATGGCTGCGAGCATGTCAAGTCCAGCAGCCTTGGCTCTTGTGGATGAGAGAATGAGCACTGAAGGAACAGGATTGCCATTTGGGTTGAGCAACAACCTTCTTGGTTGGATCCTGTTTGGTGTGTTTGGTCTGATCTGGTCTCTTTACTTTGTCTACGTTTCCGGACTTGAAGAGGACGAGGAGTCTGGATTGTCCCTGTGA

*CL2764.Contig25_All:*

ATGGCAGACGAAGCCAAAGCCAAGGGCAACGCAGCCTTCGCAGCCGGCGACTTCTCCACCGCAATCCGTCACTTCACCACTGCAATCGACCTCTCTCCGACCAATCATGTCCTCTACTCTAACCGCTCCGCCGCCCACGCTTCTCTCCACAACTACTCGGAAGCCTTAGCCGATGCTACCAAAACGGTCGAGCTCAACCCCACTTGGTCAAAAGGCTACAGCCGTCTCGGCGCCGCTCACTTGGGGTTGCACCACTACGACGACGCTGTGGCTGCGTACAAGAAGGGTCTCGGGATTGATCCCAACAATGATGGGTTGAAGTCGGGGTTAGGTGATGCGGAGGCCGCTGCGGCACGAGCGAGGAGAAGTGCGGGGCCGGGTGCGGGGGCGAGTCCGTTCGGGGATGCGTTTTCGGGGCCAGAGATGTGGGCGAAGTTGACTGCGGATCCATCCACTAGAGGGTTTCTACAGCAGCCGGATTTTGTAAAAATGATGCAGGAGATTCAGAAGAATCCGAGTTGTTTGAATTTGTATTTGAAGGATCAGAGGGTTATGCAGGCGCTTGGGGTTTTGTTAAATGTGAAGATTCATACACCAGGGTCGGGTGCTGGTGCGGAGGAGGATATGGATTCGGGGGAGGATATGGATCCGGAAGAGGTTCCGGAACCAAAGAAGGCAGAGCCAGAAAAGGCAGAGAAGGAGCCACAGCCTGAACCGATGGAGTTGAAAGAGGAGGAGAAGGAGAAGAAAGAGAGGAAGGAGCAGGCGGTTAAGGAGAAAGAGGCCGGGAATGCGGCATATAAGAAGAAGGATTTTGAGACTGCCATTCAGCATTACACTAAGGCTATGGAGTTGGATGATGCTGACATTCTGTACATTACTAACAGGGCAGCTGTTTATCTCGAGATGGGGAAGTATGATGAGTGTATTAAAGACTGTGATAAAGCGGTGGAAAGGGGTCGGGAGTTGCGTGCAGACTATAAGATGGTTGCAAAAGCCTTGACTAGGAAGGGTACTGCACTTGTAAAAATGGCAAAAACCTCTAAAGACTTCGAGCCTGCTATTGAGACTTTCCAGAAAGCTCTGACAGAGCATCGTAACCCAGACACTCTGAAGAAACTAAATGATGCTGAGAAAGCAAAGAAGGATCTTGAGCAACAAGAGTATTTTGATCCAAAGATAGCTGATGAGGAAAGAGAGAAAGGAAACGAATATTTCAAGCAACAAAATTATCCGGAAGCTGTGAAGCACTACACAGAGGCTATTAAAAGGAATCCTAAGGATCCTAAGGCATACAGTAACAGAGCTGCATGCTACACAAAACTGGGAGCACTTCCTGAGGGACTGAAGGATGCAGAGAAGTGCATTGAGCTTGAGCCAACCTTTTGTAAAGGATACAGTAGAAAAGGTGCTGTTCAGTTCTTCATGAAAGAATATGACAAAGCCATGGAAACCTACCAAGAGGGATTGAAACATGACCCAAAGAATCAGGAATTGCTAGATGGTGTACGAAGATGTGTAGAGCAAATCAACAAAGCTAGCCGTGGTGAGTTAAGCCCTGAAGAACTGAAGGAAAGACAGGCCAAGGCAATGCAGGACCCTGAAATCCAGAACATTCTAACAGACCCTGTGATGAGAGAAGTCTTGACTGATTTCCAAGAGAACCCTAGAGCTGCCCAAGAACACATGAAGAACCCCATGGTCATGGGCAAGATTCAGAAGTTAGTCAGTGCGGGGATTGTCCAGATGAGGTGA

*CL9374.contig2:*

CAAAACAACAGTCAGCAAATCAAAGAAAAAACAATGGCGTCAAGCAAAACCCAAGACAAGAAACCAAACACGAAAAACAACAAAACCAAAAACAACATAGGAAAATGCAGCACTTCAAAAACCCGACAAAAGCCGAGACAAGACGAGAACGAAACACTCCAGCAGATCGCAAAGCAAAAACATGACAAGAAGCAGAAGGAGGAGAGTAAATCCAAAGGGAAAGCTGTAGAGAGAGAAAGCAACAAGAGAGAGAAAGGTGTTGATGCGGGAGATGATGGAGAAGAGGAGACGTGCAAGTTCGCTTTCACTAGAGTGAAGAGGATCATTAAGAGTGAGCTTTCCGATATGAAGATCAGTAATGATGCTGTGTATCTCATCAACAAAGCTACTGAGAAGTTCCTTGAAACTTTTTCTCAAGATGCATATGCTTCTTCTGTGGAGGACCGCAAGAAAAGTATTAGCTACAAGCATCTATCCAAGGTTGTTCATAATGAAAAGAGATATGAGTTTCTCTCAGATTTTGTTCCAGAGATGGTAAAAGCCGAGGATGCGTTAGCCGAACGGATGAAGACTATGGATGCTTTTGGAAGTTAA

*CL9374.contig1:*

AACAACACTCAGCAAATCAAAGAAAAAACAATGGCGTCAAGCAAAACCCAAGACAAGAAACCAAACACGAAAAACAACAAAACCAAAAACAACATAGGAAAATGCAGCACTTCAAAAACCCGACAAAAGCCGAGACAAGACGAGAACGAAACACTCCAGCAGATCGCAAAGCAAAAACATGACAAGAAGCAGAAGGAGGAGAGTAAATCCAAAGGGAAAGCTGTAGAGAGAGAAAGCAACAAGAGAGAGAAAGGTGTTGATGCGGGAGATGATGGAGAAGAGGAGACGTGCAAGTTCGCTTTCACTAGAGTGAAGAGGATCATTAAGAGTGAGCTTTCCGATATGAAGATCAGTAATGATGCTGTGTATCTCATCAACAAAGCTACTGAGAAGTTCCTTGAAACTTTTTCTCAAGATGCATATGCTTCTTCTGTGGAGGACCGCAAGAAAAGTATTAGCTACAAGCATCTATCCAAGGTTGTTCATAATGAAAAGAGATATGAGTTTCTCTCAGATTTTGTTCCAGAGATGGTAAAAGCCGAGGATGCGTTAGCCGAACGGATGAAGACTATGGATGCTTTTGGAAGTTAA

*ZxACTIN:*

GTGGTCGTACAACAGGTATTGTGCTGGACTCTGGTGATGGTGTGAGTCACACTGTGCCAATATATGAAGGTTATGCCCTTCCCCATGCAATCCTTCGTCTAGACCTTGCTGGTCGTGACTTGACAGATTCTTTGATGAAGATTCTTACTGAGAGAGGGTATATGTTTACTACCACCGCTGAGCGAGAAATTGTCCGTGACATAAAGGAGAAGCTTGCATATGTTGCTCTTGACTATGAGCAAGAACTTGAACTTGCCAAAAGCAGTTCATCAGTTGAGAAGAACTATGAGCTTCCTGATGGACAGGTGATCACAATTGGGGCTGAGAGGTTCCGTTGCCCAGAAGTCCTTTTCCAGCCATCCCTTGTTGGAATGGAAGCTGTTGGAATTCATGAGACCACCTACAACTCCATCATGAAATGTGATGTGGATATCAGAAAGGATCTTTATGGAAATATTGTTCTCAGTGGTGGTTCAACCATGTTTCCTGGTATTGCAGACCGTATGAGCAAGGAGATCACTGCACTTGCTCCGAGCAGCATGAAGATCAAAGTTGTGGCTCCACCTGAAAGAAAGTACAGTGTCTGGATTGGAGGTTC

Table S12. Genes and primers used for RT-qPCR validation.

| Gene ID | Forward primer (5’→3’) | Reverse primer (5’→3’) |
| --- | --- | --- |
| *CL1054.Contig5_All* | GAGGCTGAAACGAGATAAGAATG | GCCATCCCCAATAAAGACACTG |
| *CL1594.Contig16_All* | ATGGAGGGTCTTCATGAAGGT | CCATAAGTGTTAAGCTGCCTGAC |
| *CL5761.Contig5_All* | GAGGAACGGGAAATTCTAGTG | GCACATGCCGGAACTCAATAT |
| *CL13209.Contig7_All* | GTTCTTTTGCTCCTAACTGCCC | GGCTCGACTCAAACCTGGAG |
| *CL239.Contig9_All* | TCGCTCGTGATTTGTTGCC | TTCCGTCGTTGAATGTCGC |
| *Unigene30694_All* | GGCTGAGACGCTTGCTAAATG | CAGAACCTGCAGGCATCTCT |
| *CL9799.Contig1_All* | GCACAGGACTTACGCAACC | GCTTGGCTCATCTTCTTCTCA |
| *CL1702.Contig18_All* | ACTTTACAACCGAGGGTGAGGT | CAACAGTGGTGATATGCGCTTG |
| *CL2232.Contig10_All* | AGCGTGGTGGGTTTTGATTTCG | TGGTCATTCTCAGGCAAGTCC |
| *Unigene9635_All* | AGACAGGCTACTAAGGATGCTG | CCGAGACTCAGAGGCGTAACAT |
| *Unigene33461_All* | TTGAAGAATCTCCAGCAAAGGG | GTAGCCATAACTCCTTGAAGACC |
| *CL10111.Contig12_All* | CGTCTGCTCGGGTGAAGTCT | CATCCATTGACCCCTCCGT |
| *CL12279.Contig14_All* | CTGACAAAGGCGGTGATCCTG | CTTCTACCTCCCCTACTGCTCC |
| *CL1088.Contig11_All* | GCTCCTATTATTCTTCGCCTCG | GCAACAATGTCCTGGTCGC |
| *Unigene18687_All* | CCAACACTGCTGCCTCATCTAT | AACTGATGCCCTTTCCTTTACG |
| *CL411.Contig4_All* | CAGGTGATGGTGAAGGAGGG | CATCTTCTGGCTTGACACGGT |
| *Unigene40490_All* | AACACTGCCGCCTCATCTATC | AACTGATGCCCTTTCCTTTACG |
| *CL2764.Contig25_All* | GTCCTCTACTCTAACCGCTCCG | CGCATCACCTAACCCCGACT |
| *CL9374.contig2* | AGCTACAAGCATCTATCCAAGGT | AGTCTTCATCCGTTCGGCTA |
| *CL9374.contig1* | GTTGATGCGGGAGATGATGG | GCATCCATAGTCTTCATCCG |
| *ZxACTIN* | TTTTCCAGCCATCCCTTGTT | TGCAGTGATCTCCTTGCTCATAC |
